# The Ecological-Health Risks of Potentially Toxic Metals in the Surface Sediments and Leaves of Salt-Secreting *Avicennia officinalis* as Potential Phytoremediators: A Field-Based Biomonitoring Study from Klang Mangrove Area

**DOI:** 10.3390/biology12010043

**Published:** 2022-12-26

**Authors:** Chee Kong Yap, Khalid Awadh Al-Mutairi

**Affiliations:** 1 Department of Biology, Faculty of Science, Universiti Putra Malaysia, Serdang 43400, Malaysia; 2 Department of Biology, Faculty of Science, University of Tabuk, Tabuk P.O. Box 741, Saudi Arabia

**Keywords:** *Avicennia*, metals, Klang, mangrove, ecological risk

## Abstract

**Simple Summary:**

This study evaluated the capacity for the phytoremediation of mangrove *Avicennia*, sampled from the Klang mangrove ecosystem, and analysed the ecological-health concerns of potentially toxic metals in the surface sediments. All of the hazard index values of the surface sediments for Cu, Ni, Pb, and Zn, based on a combination of three pathways, indicated 1.00, suggesting that the four metals are not carcinogenic. The lamina has more potential as a phytoremediator of vital Cu, Zn, and Fe, according to the bio-concentration factor values. As a phytoremediator of non-essential Pb and Ni, midrib plus petiole has greater potential. The data presented in this study can be used to monitor and provide ecological-health hazards of potentially toxic metals in the Klang mangrove ecosystem to lessen the threats to the ecosystem. Using the current findings to manage the Klang mangrove ecosystem, a water-energy-food framework can be proposed.

**Abstract:**

This study aimed to evaluate the ecological-health risks of potentially toxic metals in the surface sediments on the Klang mangrove ecosystem and assessed the phytoremediation potential of *Avicennia officinalis* collected from the area. The results showed that the concentrations (mg/kg dry weight) of Cu, Ni, Pb and Zn in the surface sediments ranged between 5.30–63.8, 14.2–32.7, 30.3–62.3, and 46.4–269, respectively. The ecological risk values of the surface sediments indicated that Ni, Pb and Zn were all classified as ‘low potential ecological risk’, while the Cu ecological risk ranged between ‘low potential ecological risk’ and ‘considerable potential ecological risk’. For the health risks on the sediments, all of the values of hazard index for Cu, Ni, Pb and Zn, based on a combination of three pathways, indicated < 1.00, showing that the four metals are non-carcinogenic. Based on the bioconcentration factor values, it can be concluded that the lamina has better potential as a phytoremediator of essential Cu, Zn and Fe. In contrast, midrib plus petiole has better potential as a phytoremediator of non-essential Pb and Ni. To mitigate the threats to the Klang mangrove ecosystem, the information offered in the present study can be employed in the monitoring and provision of the ecological-health risks of potentially toxic metals in the Klang mangrove ecosystem. Hence, the present findings can be employed for developing a water-energy-food framework for managing the Klang mangrove ecosystem.

## 1. Introduction

Mangrove ecosystems are significant intertidal estuarine wetlands along tropical and subtropical coasts [1,2,3]. Mangroves are a form of woody plant community and are regarded as distinct halophytes, which are an uncommon variety of evergreen trees [4]. The mangrove plants are precious to marine species as habitats, food sources, and refuges. However, the mangrove ecosystem may suffer due to anthropogenic pressure caused by population expansion. Potentially toxic metals (PTM)s are one of the factors causing a considerable detrimental effect on the ecological quality of mangroves [5,6,7]. At present, PTM contamination brought on by human activity linked to growing urbanisation and industrialisation poses a severe threat to these intertidal communities [8]. The destruction of mangroves has been linked to increased PTM concentrations in surface sediments from mangrove wetlands worldwide [9]. This destruction is usually caused by agro-based industries, industrial effluents, sewage treatment plants’ agricultural runoff, and leaching from residential rubbish dumps [4,5,7,10,11].

The mangrove ecosystem contributes to the sequestration of carbon and the prevention of coastal erosion and provides suitable feeding grounds for migratory birds. The mangrove sediment plays a significant role in limiting the movement of PTMs in estuarine ecosystems [12]. This causes the mangrove forests operate as a natural filter of wastewater from the land, helping to keep marine ecosystems in good health [13,14]. The *Avicennia* species are thought to have accumulative qualities to several PTMs and more robust tolerance [11,14,15]. Due to these distinguishing characteristics, those concerned with issues pertaining to conservation have recently begun to pay more attention to the contamination of mangrove ecosystems [16,17]. As a result, developing or enhancing conservation tactics for mangrove ecosystems is now a priority [18].

In Malaysia, *Avicennia officinalis* (Family: Avicenniaceae) is an intertidal open coast-riverine mangrove [19]. It starts flowering in January, with a decreasing population trend and is under the ‘Least concern’ conservation status [20]. The mobility of Na^+^ ions is higher in salt-secreting species (such as *Avicennia* spp.), and their concentrations in xylem sap are roughly one-tenth that of saltwater, with salt glands lowering the overall concentration of Na in leaves [21]. In addition to the processes that control Na influx/transport, mangroves likely achieve metal influx and transport regulation by many other routes.

According to Omar and Misman [22], mangrove areas in Peninsular Malaysia have been documented as 116,746, 114,353, and 110,953 ha in the years 1990, 2000 and 2017, respectively. The mangrove area in Selangor for 2017 has been estimated as 20,853 ha [22]. Therefore, there is little doubt that the KME is expected to decline in area size and quality due to many developmental activities [23].

The introduction of many contaminants, including PTMs, into the Klang mangrove ecosystem (KME) may be justified due to the effects of industrial activities and the expansion of human settlement [24]. Human activities such as logging, fisheries and tourism are among the culprits of environmental degradation in KME [17,25]. Being a highly urbanised area, the KME is also close to the busiest maritime centre in Selangor [26,27].

Although there have been some studies on the assessment of PTM levels in KME [18,28,29,30,31,32], there is little information on the ecological-health risk assessment of PTM contamination in mangrove environments or on the capacity of mangroves to accumulate and translocate PTMs within their various compartments at KME.

There have been no reported studies on the PTM in the surface sediments collected in 2007 in the KME in the literature. The present PTM data, based on the 2007 samples, are important for conducting a baseline comparison before any rehabilitation project to clean the river has been conducted after 2007. However, there is a strong possibility that the KME could be polluted to an even higher rate due to human expansion and industrialization [17,25]. Therefore, the 2007 data on KME could lead to more comprehensive environmental management in order to understand the condition of its ecosystem in 2007 and to provide the data for future monitoring. Thus, the objectives of this study were to: (1) evaluate the ecological-health risks of PTMs in the surface sediments on the KME; and (2) assess the phytoremediation potential of PTMs in *A. officinalis* collected from the KME.

## 2. Materials and Methods

### 2.1. Sampling Site Descriptions

**Figure 1:**
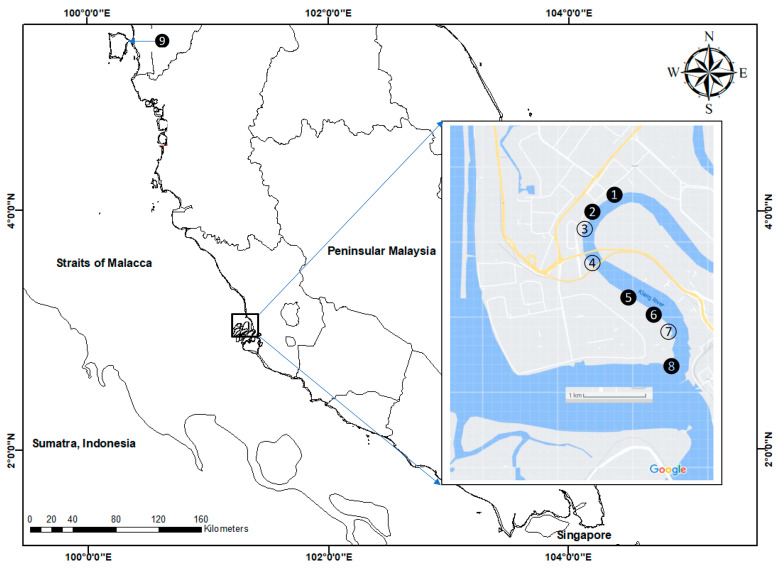
Sampling sites for surface sediments in the Klang mangrove ecosystem (S1–S8) and Juru mangrove ecosystem (S9) from the present study. Leaves of mangrove *Avicennia* were collected at the blacken circles (S1, S2, S5, S6, S8 and S9).

The sediment samplings were conducted in the estuary section of the Klang River with the mangrove ecosystem (S1–S8) on 2 December 2007, while sediment samplings from the site at Juru mangrove (S9) were collected on 8 December 2007, as can be seen in Figure 1 (Table S1). The sampling site at Juru (S9) was selected because it was the previously reported known polluted site [33]. Hence, it is important for reference purposes. In all of the sampling sites (S1–S9), three subsamples of surface sediments in the mangrove area were collected using a clean plastic scoop. However, the leaves of mangrove *Avicennia* were hand-collected at 6 sites (S1, S2, S5, S6, S8 and S9) (Figure 1). At every sampling site, 20 old leaves from three different trees of *Avicennia* were collected. Upon collection, the samples were transferred to the laboratory in zipped-lock polyethylene bags.

All of the samples were brought to the laboratory and oven-dried at 80 °C for 72 h to constant dry weight. All of the dried sediments were passed through a 63 µm sieve. The leaves samples were separated into the lamina and the midrib plus petiole (MP). The dried leaves were ground to homogenize using a pestle and mortar.

### 2.2. Metal Analysis

For the oven-dried plant samples, 5 mL of concentrated nitric acid (HNO_3_, AnalaR grade, BDH 69%) was added to the samples [34]. For the oven-dried sediments, each sample was digested using a combination of concentrated nitric acid (HNO_3_, AnalaR grade, BDH 69%) and perchloric acid (HClO_4_, AnalaR grade, BDH 60%) in the ratio 4:1 (10 mL) [35,36,37]. The digestion tubes with samples were put into a digestion block at 40 °C for 1 h, and then at 140 °C for 3 h [35,36,37]. They were then diluted to 40 mL with double de-ionised water. Later, the diluted samples were filtered through Whatman No. 1 (filter speed: medium) filter paper into acid-washed pillboxes until metal determination.

The sediment samples were fractionated into four fractions, namely: (i) ‘Easily, freely, leachable, or exchangeable’ (F1); (ii) ‘Acid-reducible’ (F2), (iii) ‘Oxidisable-organic’ (F3); and (iv) ‘Resistant’ (F4), according to Badri and Aston [38]. The total concentrations are the summation (SUM) of all the above four geochemical fractions.

All of the samples were analysed for Zn, Ni, Fe, Pb and Cu using an air-acetylene flame atomic absorption spectrophotometer (FAAS, Perkin Elmer Model AAnalyst 800; Perkin Elmer LLC, CT, USA). Standard solutions were prepared from the stock solution provided by MERCK Titrisol for the five metals, and the data were presented on an mg/kg dry weight basis.

For quality control and quality assurance, all glassware and non-metal apparatuses used in this study were soaked in an acid bath (5% HNO_3_) for 72 h after being washed with laboratory-grade detergent (Decon 90) to avoid possible contamination. The apparatuses were washed and soaked in laboratory-grade detergent (Decon 90) for at least 3 h before the analysis. Procedural blanks were employed, and the quality control samples were made by diluting the standard solutions of the metals to be tested. These standard solutions were analysed after every 5–10 samples to check the accuracy of the analysed samples.

Four types of Certified Reference Materials (CRMs) were checked with the samples to ensure the accuracy of the FAAS measurements. These CRMs included Dogfish Liver-DOLT-3 (National Research Council Canada), *Lagarosiphon major* (NR.60), and NSC DC 73,319 (soil), and marine sediments-(MESS-3, National Research Council Canada, Beaufort Sea), Their recoveries were mostly acceptable (between 70–120%) (Table S2). The detection limits for Fe, Cu, Ni, Pb, and Zn were 0.010, 0.010, 0.010, 0.009, and 0.007 mg/L, respectively.

### 2.3. Ecological Risk Assessments

#### 2.3.1. Geoaccumulation Index

The geoaccumulation index (I_geo_) was used to determine the degree of metal pollution in the area. The calculation of I_geo_ was based on Equation (1) [39].

(1) $$I_{\mathrm{geo}}=\log_{2}\frac{\mathrm{Sample}}{1.5\times \mathrm{Background}}$$

where ample is the concentration measured; the background is the background concentrations in the present study, based on the concentrations (mg/kg dry weight (dw)): 9.48, 3.55, 7.31, and 13.16 for Pb [40], Cu [41], Ni [42], and Zn [43], respectively, based on the intertidal area of Peninsular Malaysia (Table S3).

The value (1.5) is the correction factor to mitigate the lithogenic effluents. There are six established classifications of pollution that are well described by Muller [39].

#### 2.3.2. Ecological Risk Index

Firstly, the contamination factor (CF) calculation was based on the pollution of a single metal factor in Equation (2).

(2) $$\mathrm{CF}=\frac{C_{s}}{C_{B}}$$

where C_s_ is the concentration of PTM in the surface sediments; C_B_ is the background values based on the intertidal area of Peninsular Malaysia, as mentioned above.

Later, the ecological risk (ER), the potential ER of a single element, was calculated based on Equation (3).

(3) $$\mathrm{ER}=T_{R}\times \mathrm{Cf}$$

where T_R_ is the toxic response factor of a single element. The T_R_ values used in the present study are Cu = 5.00, Ni = 5.00, Pb = 5.00, and Zn = 1.00 [44]. CF is the contamination factor calculated, as described in Equation (1). There are 5 classifications of ER based on Hakanson [44].

### 2.4. Human Health Risk Assessment

The human health risk assessment (HHRA) of the surface sediments is calculated to determine the non-carcinogenic risk (NCR) to humans by using exposure pathways through inhalation, ingestion, and dermal contact. The estimation of the HHRA followed the public-domain guidelines in the Exposure Factors Handbook of the US Environmental Protection Agency [45,46,47,48]. The average daily doses (ADDs) (mg/kg day) of PTMs through inhalation (ADD_inh_), ingestion (ADD_ing_), and dermal contact (ADD_der_) for both children and adults were calculated by using Equations (4)–(6), as follows:

(4) $${\mathrm{ADD}}_{\mathrm{inh}}=C_{\mathrm{sediment}}\left(\frac{\mathrm{InghR}\;\times \mathrm{EF}\;\times \mathrm{ED}}{\mathrm{PEF}\;\times \;\mathrm{BW}\;\times \;\mathrm{AT}}\right)\;$$

(5) $${\mathrm{ADD}}_{\mathrm{ing}}=C_{\mathrm{sediment}}\left(\frac{\mathrm{IngR}\;\times \mathrm{EF}\;\times \mathrm{ED}}{\mathrm{BW}\;\times \;\mathrm{AT}}\right)\times 10$$

(6) $${\mathrm{ADD}}_{\mathrm{der}}=C_{\mathrm{sediment}}\left(\frac{\mathrm{SA}\;\times \mathrm{AF}\;\times \;\mathrm{ABS}\;\times \mathrm{EF}\;\times \mathrm{ED}}{\mathrm{BW}\;\times \;\mathrm{AT}}\right)\times 10$$

where ADD_inh,_ ADD_ing_, and ADD_der_ are the daily amounts of exposure to metals (mg/kg day) through inhalation, ingestion, and dermal contact, respectively. The definition, reference values, and exposure factors employed to estimate the intake values and health risks of PTMs in sediments are given in Table S4.

In this study, the NCR of PTMs was assessed through the hazard quotient (HQ) and hazard index (HI), as used in the literature [49,50]. The HQ is the proportion of the ADD of a metal to its reference dose (RfD) for similar exposure pathway(s) [35,48]. The RfD (mg/kg day) values of all metals used in the present study are given in Table S4.

The NCR is assessed by HI, which is the summation of the HQs in the three exposure pathways [51,52,53,54]. The HI was calculated according to Equation (7).

(7) $$\mathrm{HI}=\sum {\mathrm{HQ}}_{i}=\sum \left(\frac{{\mathrm{ADD}}_{i}}{{\mathrm{RfD}}_{i}}\right)$$

### 2.5. Calculation of Bioconcentration Factor

The plant’s capacity to absorb and tolerate PTMs was calculated using the bioconcentration factor (BCF). This indicator is frequently used to assess whether plants would make effective phytoremediators [55,56]. The plant’s capacity to bioaccumulate metals from sediments is assessed using BCF, in which the metal concentrations in the sediments are represented by F1, F2, F3, F4, and SUM, while the metal concentrations in the mangrove leaves are MP or lamina. Therefore, in the present study, five BCF values are used, as defined in the following Equations (8)–(12):

(8) $$\mathrm{BCF}1=\frac{{\mathrm{Plant}}_{}}{{\mathrm{Sediment}}_{F1}}$$

(9) $$\mathrm{BCF}2=\frac{{\mathrm{Plant}}_{}}{{\mathrm{Sediment}}_{F2}}$$

(10) $$\mathrm{BCF}3=\frac{{\mathrm{Plant}}_{}}{{\mathrm{Sediment}}_{F3}}$$

(11) $$\mathrm{BCF}4=\frac{{\mathrm{Plant}}_{}}{{\mathrm{Sediment}}_{F4}}$$

(12) $$\mathrm{BCF}5=\frac{{\mathrm{Plant}}_{}}{{\mathrm{Sediment}}_{\mathrm{SUM}}}$$

### 2.6. Statistical Analysis

The graphical histograms and overall statistics of the present data were obtained using Kaleida Graphs, version 5.0 (1986–2022 by Synergy Software, Eden Prairie, MN, USA). Although there are alternative methods for confirming normality, the Shapiro-Wilk test was chosen because it is the most popular and extensively used method and has a higher sensitivity to detect non-normality when the sample size is small (N < 50) [57,58,59].

All of the data were determined to have significant values for the Shapiro-Wilk Test <0.05, according to the Shapiro-Wilk normality test, suggesting that the data deviated considerably from a normal distribution. Prior to conducting multiple linear (forward) stepwise regression analysis (MLSRA) and cluster analysis, they were converted using the log_10_[value + 1] formula (CA). This log_10_-transformation was used to stabilise the variance and the lack of normality to produce a frequency distribution that was more akin to a normal distribution and to satisfy the need of normality for the regression model [60,61]. The MLSRA and CA were performed using STATISTICA (Version 10; StatSoft. Inc., Tulsa, OK, USA, 1984–2011). This has been shown by many studies on relationships between a dependent variable and independent variables [62,63,64,65,66].

For CA, the clustering patterns of the eight sampling sites of Cu, Ni, Pb and Zn in the sediments based on I_geo_, CF, and ER, and the geochemical fractions of the sediments (F1, F2, F3, F4 and SUM) were performed.

For the MLSRA based on the sediments, the HI and ER acted as the dependent variables, while the pathways of HQ_ing_, HQ_inh_, and HQ_der_, and the ecological indexes (I_geo_, CF, and ER) and geochemical fractions (F1, F2, F3, F4, SUM) of the sediments acted as the independent variables. For the MLSRA based on the *Avicennia* leaves, the lamina and MP acted as the dependent variables, while BCF1, BCF2, BCF3, BCF4, BCF5, and the sedimentary geochemical fractions (F1, F1, F3, F4 and SUM) acted as the independent variables.

## 3. Results

### 3.1. Ecological-Health Risk Assessments of Surface Sediments

#### 3.1.1. Total Concentrations of PTM and Ecological Risks Indices

**Figure 2:**
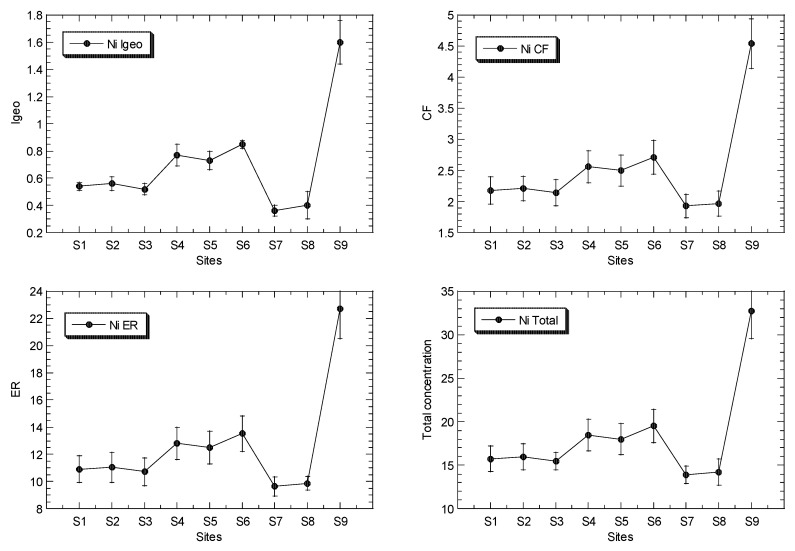

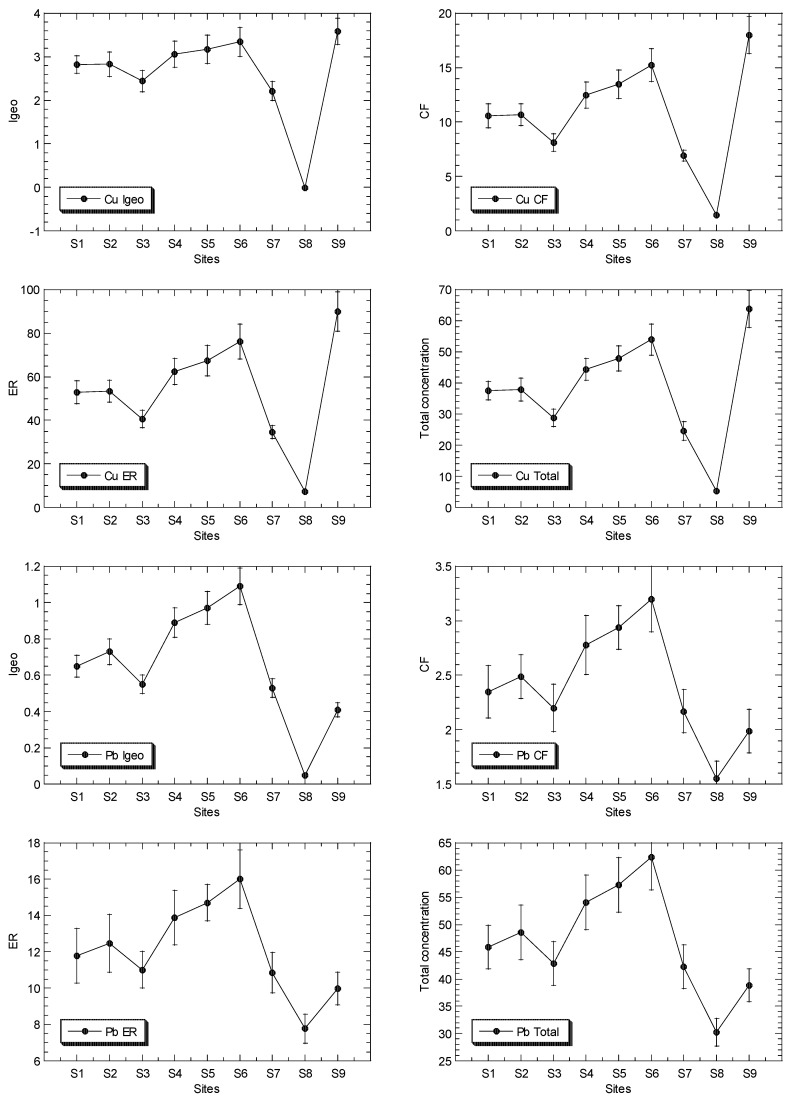

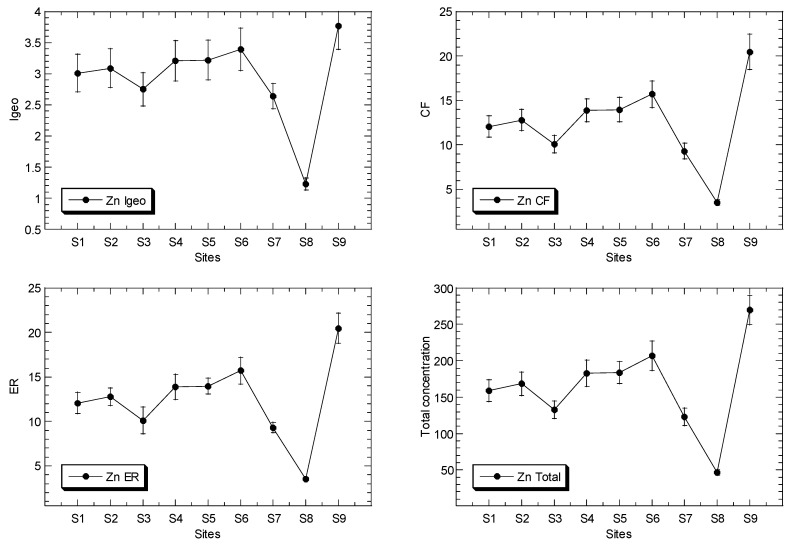
Total concentrations (mean ± standard error, mg/kg dry weight), values of geoaccumulation index (I_geo_), contamination factor (CF), and ecological risk (ER) for Cu, Ni, Pb and Zn, of surface sediments in Klang mangrove ecosystem (S1–S8) and Juru mangrove (S9), based on background concentrations (mg/kg dry weight) of the metals that were reported from Peninsular Malaysia, namely: 9.48, 3.55, 7.31, and 13.16 for Pb [40], Cu [41], Ni [42], and Zn [43], respectively.

The mean concentrations of Cu, Ni, Pb and Zn in the surface sediments of the mangrove ecosystem in the Klang River estuary and a site in the Juru estuary are presented in Figure 2, while the overall statistics are presented in Table 1, and their geochemical fractions are given in Table S5.

The total Zn concentrations in the surface sediments ranged between 46.4 and 269 mg/kg dry weight. In comparison to the SGQ values (Table S3), all of the maximum Zn levels at Juru were below those by Long et al. [67] for ERM, Macdonald et al. [68] for PEL, and Chapman et al. [69] for ISQV-high, but higher than those of Long et al. [67] for ERL, Macdonald et al. [68] for TEL, and Chapman et al. [69] for ISQV-low. However, all of the sites of KME recorded values below the SGV values. When compared these results to the reference background values (Table S3), the maximum Zn level at the Juru site recorded higher values than those of all of the SGQ values, including the background value of Zn from the west coast of Peninsular Malaysia (WCPM) [43] and the mangrove sediment from the WCPM [34]. However, the sites from the Klang mangrove generally recorded values lower than, or equal to, those of all the reference background values.

The total Fe concentrations in the surface sediments ranged between 22,121 and 27,906 mg/kg dry weight. When comparing to the SGQ values (Table S3), the Fe ranges were within the only UCC level reported by Wedepohl [70].

The total Pb concentrations in the surface sediments ranged between 30.3 and 62.2 mg/kg dry weight. In comparison to the SGQ values (Table S3), all of the maximum Pb levels at Juru were higher than those by Long et al. [67] for ERL, and Macdonald et al. [68] for TEL, but lower than those of Long et al. [67] for ERM, Macdonald et al. [68] for PEL, and Chapman et al. [69] for both ISQV-low and ISQV-high. However, all of the sites of KME recorded values below the Pb SGV values. When compared to the reference background values (Table S3), the maximum Zn level at the Juru site recorded values higher than those of the pre-industrial reference level by Hakanson [44], but higher than all of the SGQ values, including the background value of Zn from the WCPM [40] and within those of the mangrove sediment from the WCPM [34]. However, the sites from KME generally recorded lower or within those of all of the reference background values of Pb.

The total Cu concentrations in the surface sediments ranged between 5.29 and 63.8 mg/kg dry weight. In comparison to the SGQ values (Table S3), all of the maximum Cu levels at Juru ere higher those by Long et al. [67] for ERL, and Macdonald et al. [68] for PEL, and Chapman et al. [69] for both ISQV-low and ISQV-high. However, all of the sites of KME recorded values below the Cu SGV values. When compared to the reference background values (Table S3), the maximum Cu level at the Juru site recorded values higher than the SGQ values, including the background value of Cu from the WCPM [41] and the mangrove sediment from the WCPM [34]. However, the sites from KME generally recorded lower or within the Cu reference background values (Table S3).

The total Ni concentrations in the surface sediments ranged between 14.2 and 32.7 mg/kg dry weight. In comparison to the SGQ values (Table S3), all of the maximum Ni levels at Juru were higher than those by Long et al. [67] for ERL, and Macdonald et al. [68] for TEL, but lower than those by Chapman et al. [69] for ISQV-low, Long et al. [67] for ERM, and Macdonald et al. [68] for PEL. However, all of the sites of KME recorded values below or within the Ni SGV values. When compared to the reference background values (Table S3), the maximum Ni level at the Juru site recorded values higher than those of UCC by Wedepohl [70], the background value of Ni from the WCPM [42] and the mangrove sediment from the WCPM [34]. However, the sites from KME generally recorded values lower or within those of all the Ni reference background values (Table S3).

The overall values of I_geo_, CF, and ER for Cu, Ni, Pb and Zn, in the surface sediments from the Klang River estuary and the site in the Juru estuary, based on the background levels of the metals as reference a metal or normalizer that were reported from Peninsular Malaysia, are presented in Table 2 (Table S6), and the mean values are presented in Figure 2.

The I_geo_ values of Cu, Ni, Pb and Zn ranged between −0.01 and 3.58, 0.36 and 1.60, 0.05 to 1.09, and 1.23 and 3.77, respectively (Table 2). This indicated that the classifications of Cu and Zn ranged between ‘practically unpolluted’ (<0)’ and ‘strongly polluted’ (3–4), while Ni and Fe are classified between ‘unpolluted’ (0–1), and ‘moderately polluted’ (1–2), based on Muller [39].

The CF values of Cu, Ni, Pb and Zn ranged between 1.49 and 18.0, 1.93 and 4.54, 1.55 and 3.20, and 3.53 and 20.5, respectively (Table 2). The ER values of Cu, Ni, Pb and Zn ranged between 7.45 and 89.9, 9.64 and 22.7, 7.77 and 16.0, and 3.53 and 20.5, respectively (Table 2). The ER values indicate that Ni, Pb and Zn were classified as ‘low potential ecological risk’ (ER < 40)’, while Cu ranged between ‘low potential ecological risk’ (ER < 40)’ and ‘considerable potential ecological risk’ (80 ≤ ER < 160)’, according to Hakanson [44].

**Figure 3:**
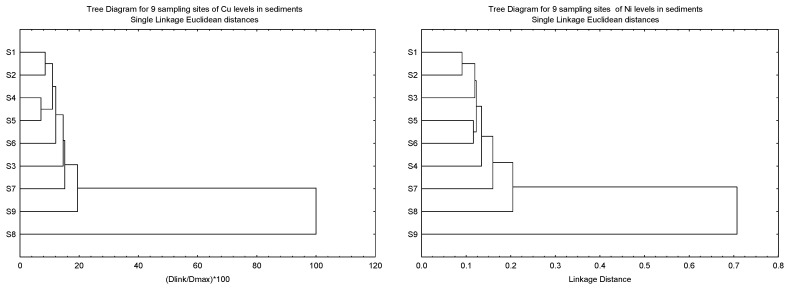

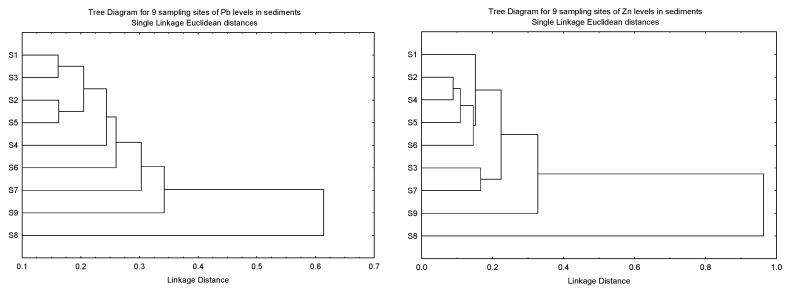
Clustering patterns of the nine sampling sites of Cu, Ni, Pb and Zn in the sediments based on I_geo_, CF, and ER, and the geochemical fractions of the sediments (F1, F2, F3, F4 and SUM). All values have been log_10_ (value + 1) before cluster analysis; Sampling sites’ information followed those in Table S1.

The clustering patterns of the nine sampling sites of Cu, Ni, Pb and Zn in the sediments based on I_geo_, CF, and ER, and the geochemical fractions of the sediments, are given in Figure 3. It was found that S8 was clustered as a single major entity from the other subclusters for Cu, Pb and Zn due to the levels of the three metals being significantly lower than the other eight sites. However, the significantly higher levels of Ni in S9 resulted in S8 being clustered as a major entity. Although the highest levels of Cu and Zn were found in S9, this site was not significantly higher than the other sites.

The outputs of the multiple stepwise regression analytical outputs, based on ER as a dependent variable and independent variables, are the hazard quotient pathways and geochemical fractions of the sediments, and are presented in Table 3. CF and HI-A were selected as the influential factors for the Cu ER. Three variables (CF, HQ_der_-C and F2) were selected as the influential factors for the Pb ER. Five variables (F1, F2, F3, F4 and SUM) were selected as the influential factors for the Zn ER. CF and HQ_ing_-C were selected as the influential factors for the Ni ER (Table 3).

#### 3.1.2. Health Risk Assessments of Surface Sediments

The values of HQ and HI in the three exposure routes of Ni in the Klang River estuary and the site in the Juru estuary are presented in Table S7, while their overall values are given in Table 4 The Ni HI values for children and adults are 9.16 × 10^−3^ to 2.16 × 10^−2^ and 1.77 × 10^−3^ to 4.18 × 10^−3^, respectively. All of these HI values indicated < 1.00, showing non-carcinogenic risks of Ni.

The values of HQ and HI in the three exposure routes of Cu in the Klang River estuary and the site in the Juru estuary are presented in Table S8, while their overall values are given in Table 5. The Cu HI values for children and adults are 1.74 × 10^−3^ to 2.10 × 10^−2^ and 2.56 × 10^−4^ to 3.09 × 10^−3^, respectively. All of these HI values indicated < 1.00, showing non-carcinogenic risks of Cu.

The values of HQ and HI in the three exposure routes of Pb in the Klang River estuary and the site in the Juru estuary are presented in Table S9, while their overall values are given in Table 6. The Pb HI values for children and adults are 1.13 × 10^−1^ to 2.33 × 10^−1^ and 1.81 × 10^−2^ to 3.73 × 10^−2^, respectively. All of these HI values indicated < 1.00, showing non-carcinogenic risks of Pb.

The values of HQ and HI in the three exposure routes of Zn in the Klang River estuary and the site in the Juru estuary are presented in Table S10, while their overall values are given in Table 7. The Zn HI values for children and adults are 2.04 × 10^−3^ to 1.19 × 10^−2^ and 3.14 × 10^−4^ to 1.82 × 10^−3^, respectively. All of these HI values indicated < 1.00, showing non-carcinogenic risks of Zn.

The outputs of the MLSRA outputs based on HI as the dependent variable and independent variables are the hazard quotient pathways and geochemical fractions of the sediments; these are also presented in Table 3. For Cu, two (HQ_ing_-C and F2) and three (HQ_inh_-A, I_geo_ and CF) variables were selected as influential factors in the HI-C and Hl-A, respectively. For Pb, seven (HQ_ing_-C, SUM, F1, F3, F2, I_geo_ and F4) and three (HQ_ing_-A, ER and F1) variables were selected as influential factors in the HI-C and Hl-A, respectively. For Zn, four (HQ_ing_-C, F4, F1 and I_geo_) and two (HQ_inh_-A and F1) variables were selected as influential factors in the HI-C and Hl-A, respectively. For Ni, two variables (HQ_der_-C and I_geo_) and HQ_der_-A were selected as influential factors in the HI-C and Hl-A, respectively.

### 3.2. Phytoextraction Potentials of Mangrove Leaves

#### 3.2.1. Zinc

The Zn levels (mg/kg dry weight) in the MP and lamina are 16.4–36.2 and 17.6–41.1, respectively (Table 8; Table S11). The mean Zn values indicated that the Zn lamina (26.9) is significantly (*p* < 0.05) higher than that (22.9) in the MP.

The overall mean values in the MP for BCF1, BCF2, BCF3, BCF4, and BCF5 are 1.10, 0.68, 0.53, 0.43, and 0.15, respectively (Table 8; Table S11). These BCF values are lower than those in lamina, in which their values of BCF1, BCF2, BCF3, BCF4, and BCF5 are 1.37, 0.82, 0.62, 0.51, and 0.18, respectively. This indicated that lamina has a better potential as a phytoremediator of Zn.

The outputs of the MLSRA outputs based on lamina and MP as dependent variables and independent variables are the bioconcentration factors and sedimentary geochemical fractions, as presented in Table 9. For Zn, BCF4 and F1 were similarly selected as influential parameters for the Zn accumulation of both lamina and MP. However, there are four variables (F4, BCF5, BCF1 and BCF2) were selected for lamina only, while only F3 was selected for MP.

#### 3.2.2. Iron

The Fe levels (mg/kg dry weight) in MP and lamina are 45.8–193, and 92.9–244, respectively (Table 10; Table S11). The mean Fe values indicated that the Fe lamina (166) is significantly (*p* < 0.05) higher than that (117) in MP.

The overall mean values in the MP for BCF1, BCF2, BCF3, BCF4, and BCF5 are 0.463, 0.117, 0.050, 0.005, and 0.004, respectively (Table 10; Table S11). These BCF values are lower than those in lamina, in which their values of BCF1, BCF2, BCF3, BCF4, and BCF5 are 0.647, 0.163, 0.073, 0.007, and 0.006, respectively. This indicated that lamina has a better potential as a phytoremediator of Fe.

For Fe, it was found that BCF5 and F4 are selected as the influential variables for the Fe accumulation of both lamina and MP. However, only BCF2 was selected for lamina while F2, BCF4 and SUMM were selected for MP.

#### 3.2.3. Lead

The Pb levels (mg/kg dry weight) in MP and lamina are 6.17–23.7, and 3.39–20.6, respectively (Table 11; Table S11). The mean Pb values indicated that the Pb lamina (10.9) is lower than that (11.92) in MP.

The overall mean values in the MP for BCF1, BCF2, BCF3, BCF4, and BCF5 are 12.8, 15.8, 0.62, 1.26, and 0.32, respectively (Table 11; Table S11). These BCF values are comparable to those in lamina, in which their values of BCF1, BCF2, BCF3, BCF4, and BCF5 are 11.4, 13.5, 0.58, 1.61, and 0.32, respectively.

For Pb, six variables (BCF3, F3, F1, BCF5, BCF1 and SUM) were similarly selected as influential parameters for the Pb accumulation of both lamina and MP. However, only BCF4 and F4 were selected for lamina, while F2 and BCF2 were selected for MP.

#### 3.2.4. Copper

The Cu levels (mg/kg dry weight) in MP and lamina are 3.77–11.6, and 5.56–11.9, respectively (Table 12; Table S11). The mean Cu values indicated that the Cu lamina (7.76) is significantly (*p* < 0.05) higher than that (6.88) in MP.

The overall mean values in the MP for BCF1, BCF2, BCF3, BCF4, and BCF5 are 7.33, 317, 63.4, 0.26, and 0.21, respectively (Table 12; Table S11). These BCF values are lower than those in lamina, in which their values of BCF1, BCF2, BCF3, BCF4, and BCF5 are 10.1, 381, 125, 0.35, and 0.30, respectively. This indicated that lamina has a better potential as a phytoremediator of Cu.

For Cu, three variables (F1, BCF1 and F3) were similarly selected as influential parameters for the Cu accumulation of both lamina and MP. However, only F4 was selected for lamina, while four variables (BCF4, BCF2, BCF5 and F2) were selected only for MP.

#### 3.2.5. Nickel

The Ni levels (mg/kg dry weight) in MP and lamina are 0.21–4.09, and 0.13–2.86, respectively (Table 13; Table S11). The mean Ni values indicated that the Ni lamina (1.74) is significantly (*p* < 0.05) lower than that (2.07) in MP.

The mean values in the MP for BCF1, BCF2, BCF3, BCF4, and BCF5 are 3.59, 1.70, 0.32, 0.25, and 0.13, respectively (Table 13; Table S11). These BCF values are higher than those in lamina, in which the values of BCF1, BCF2, BCF3, BCF4, and BCF5 are 2.84, 1.41, 0.27, 0.21, and 0.10, respectively. This indicated that MP has a better potential as a phytoremediator of Ni.

For Ni, three variables (BCF3, BCF1 and BCF4) were similarly selected as influential parameters for the Ni accumulation of both lamina and MP. However, four variables (BCF5, BCF2, F2, and F1) were selected for lamina only, while F3, F4 and SUM were selected only for MP.

## 4. Discussion

### 4.1. Mangrove Leaves of Avicennia Officinalis Have Low Metal Concentrations

The low level of detected metal accumulation in the mangrove leaves could be explained by a variety of different mechanisms, as indicated in the differences in the influential parameters selected for the accumulation of metals between lamina and MP. In estuarine environments, the initial bioavailability of sediment metals is frequently poor. Metals frequently precipitate as insoluble sulphides in sediments because sediments are typically anoxic, waterlogged, and have a low pH [71]. Metals may be integrated into the lattice structure of clay, adsorbed on the ion exchange sites of fine silts and/or clays, or adsorbed inside iron and manganese colloidal oxide complexes [72]. Detritus-rich sediments’ high organic content promotes complexation with refractory organics, which significantly lowers metal availability [73]. High salinity also promotes the development of metal-chloride complexes, which are less bioavailable for absorption than free metals [74].

Processes in the root’s rhizosphere are among the physiological systems that may be of the cause of the variation in the uptake and translocation at the root level. The absence of these adaptations in some species and the adaption of aerial root structures in others, such as pneumatophores with lenticels for gaseous exchange, may account for the small-scale variations in uptake within families and genera. Higher concentrations of some trace metals in the exchangeable form may come from an oxidised rhizosphere’s ability to diminish the stability of iron plaques and reduce complexing sulphides in anoxic soil environments [75,76]. In fact, trends toward increased root BCFs appear to be specific to species that have pneumatophores of *Avicennia*.

The present survey’s findings of the restricted translocation of Cu, Zn, and particularly Pb between species and the accumulation of Cu, Pb, and Zn by roots (root BCFs 1) are consistent with the patterns of accumulation and distribution discovered in *A. marina*’s laboratory experiments. Cu, Pb, and Zn were discovered in *A. marina* roots at quantities equivalent to the sediment loadings reported by MacFarlane and Burchett [15,77]. The metals could be primarily concentrated in cell walls, such as apoplastic transport implicated through cationic exchange with cell wall-associated carboxylic groups. The endodermis prevented the entry of Cu, Pb, and Zn into the stele, while the epidermal layers served as a barrier to reduce the transfer of Pb.

It was reported by MacFarlane and Burchett [15,77] that Pb transfer to leaves in *A. marina* was minimal. The Pb accumulation in the root was mainly immobile, cell wall bound, and/or sequestered in the epidermal layers. Zinc and Cu were translocated to the leaf tissue; however, the concentrations were much lower than in roots (TF < 0.1) and it is likely that they were chelated with organic acids. Despite the variations in the salt management mechanisms, these patterns of translocation do in fact largely replicate the cross-species trends. The secretion of metals does not significantly change the overall distribution patterns of metals in leaf tissue among families, or when comparing secretors to non-secretors, despite the fact that mangroves have been shown to excrete metals (Cu and Zn) in *A. marina* [15].

The BCF values in mangroves are relatively modest when compared to well-established hyperaccumulators, such as the fern *Pteris vittata* [78], which has As BCF levels greater than 100. Other hyperaccumulating terrestrial plant species, such as *Thlaspi caerulescens*, have leaf BCFs for Zn that are up to 40, which are lower than those for As [79]. These results are typically shown as absolute values rather than as BCF or TF. According to Brooks [55], plants that accumulate more than 500 mg/kg of copper or 10,000 mg/kg of zinc in their tissue are categorised as hyperaccumulators. *Brassica juncea*, a Pb hyperaccumulator, has been found to accumulate 18.8 mg/kg of Pb in its shoots [80]. Mangroves demonstrate metal uptake and translocation at rates that are significantly lower than those of established hyperaccumulators. As long-term sinks for metallic pollutants, mangroves are therefore perhaps best used in cleanup efforts as phytostabilizers [81]. Not only can mangrove sediments decrease numerous metals in anoxic sediments, limiting their bioavailability and mobility, but mangrove trees also physically stabilise the sediments, preventing the export of sedimentary metals to nearby waterways.

Based on the mangroves in Sundarban, Chakraborty et al. [56] reported the concentration (mg/kg) of Zn was 18.54–28.2 in the leaves, that of Cu was 6.76–11.9 in the leaves, and that of Pb was 3.26–4.57 in the leaves of *A. officinalis* in Jharkhali (India), They concluded that the mangrove leaves had the phytoremediation capacity of the PTMs. Ghosh et al. [82] found significant correlations between the antioxidative enzyme activity, photosynthetic pigments and PTM levels. This indicated the active functioning of the PTM detoxification mechanism in *Avicennia officinalis*. Later, Ghosh et al. [83] found that low BCF in the mangrove rhizosphere observed in the river Hooghly might be due to a barrier to hypodermal structures and/or any prevailing saturation mechanism of HMs. Kholoud et al. [84] found the metal concentrations in the leaves of *A. marina* at three sites along Tubli Bay were in the following order: Fe > Zn > Cu > Ni.

### 4.2. The Mangrove of Avicennia Officinalis to Act as a Potential Phytoremediator of Potentially Toxic Metals

There are two points to support the above statement. Firstly, the PTMs in the habitat surface sediments of *A. officinalis* had low ER; secondly, there are no non-carcinogenic risks of Ni, Cu, Pb and Zn in the surface sediments regarding the differences of the influential parameters selected for the PTMs’ HI values in the sediments between children and adults. In general, Cu and Cr had the greatest BCF values across all of the sites that were studied, indicating that these two metals were significantly bio-accumulated in *A. marina* and had higher mobility than the other metals that were also under investigation.

The low bioavailability of metals in sediments and/or preventing metal uptake by mangroves can account for the lowest BCF values found in highly metal-contaminated sediments. It has been hypothesised that by complexing with organic matter and/or precipitating sulphides under decreasing conditions, mangrove sediments can immobilise metals in inaccessible forms [73,85]. Therefore, more research is needed on speciation and the readily accessible metal components in mangrove sediments. Usman et al. [2] found that the majority of these BCF values were deemed to be excessive (>1), indicating that *A. marina* may be a highly effective plant for the bioaccumulation of PTMs.

### 4.3. The Lamina Has a Better Potential as a Phytoremediator of Essential Cu, Zn and Fe, While the Midrib plus Petiole has a Better Potential as a Phytoremediator of non-essential Pb and Ni

The essential metals (Cu and Zn; leaf BCFs of 0.47 and 0.51, respectively) demonstrated better mobility than the non-essential metals, according to a review by MacFarlane et al. [18] (Pb; leaf BCF of 0.11). Mangroves generally act as regulators of necessary metals, such as Cu and Zn, and exclude species for non-essential metals, such as Pb [86]. The results of the MLSR with all of the differences in the influential variables being selected for the accumulations of Cu, Zn, Fe, Pb and Ni clearly indicated that the phytoremediation strategies for the five metals were dissimilar.

Cu and Zn, two important elements, saw a decline in leaf BCF when the environmental concentrations rose. These patterns may indicate that plants move metal from their roots to their shoots to meet their metabolic needs at low metal concentrations, while at higher concentrations, metal translocation is controlled to prevent toxicity [86]. Regardless of environmental concentrations, non-essential elements such as Pb are not present in the leaf tissue.

Arumugam et al. [87] reported that the concentration of heavy metals in the samples of *A. marina* collected from the Muthupet mangrove forest had increased, particularly non-essential metals such as Cd and Pb. Compared to non-essential metals, essential elements, such as Cu and Zn, were more abundant in the sediment of the rhizosphere of *A. marina*. As a result, the plant was using Cu and Zn for their active metabolism. Alzahrani et al. [3] collected twenty-one sets of sediment samples and pieces of mangroves along the Red Sea coast of Saudi Arabia to evaluate the concentration PTMs and found that Cu > Ni > Pb where the metals with the highest mean concentrations in the sediments. The BCF values were > 1, indicating that *A. marina* can accumulate PTMs, particularly Pb. Based on *A. marina* L. collected from Rabigh lagoon, Aljahdali and Alhassan [9] found that all of the metals had a BCF of >1.00, and antioxidants and Pb had a positive connection. This could be due to *A. marina*’s capacity to exclude or detoxify Pb through its mechanism of exclusion or detoxification.

Souza et al. [88] evaluated the accumulation and translocation of metals in *Avicennia schaueriana* from the sediment to the roots and leaves. Plants thriving in less contaminated environments had the highest BCF values. In contrast, plants from the most polluted areas are had the highest translocation factors, showing that *A. schaueriana* could stand the unfavourable circumstances. In other words, when exposed to high amounts of metals in the environment, the roots’ ability to absorb metals is reduced; instead, plants appear to improve their metal translocation to lower the concentration of harmful metals in the roots. They suggested that *A. marina* may be categorised as a potential phytoextraction agent for Cu.

According to Usman et al. [2], the amount of PTM accumulation varied depending on the type of metals and the sections of *A. marina*. In the order Cu > Zn > Cr > Ni > Cd, rather high levels of PTMs were found in mangroves. The highest levels of Cu in *A. marina* are correlated with the highest levels of Cu in the nearby sediments. The fact that Cu and Zn are crucial trace elements for plants may help to explain the high amounts of these two metals.

Similar to our findings, earlier research conducted with *A. marina* revealed that Pb showed little absorption and minimal movement, while Cu and Zn had the largest accumulation [15]. The lower Pb accumulation in mangrove leaves could be due to Pb having a limited solubility and is extremely immobile, at pH levels above 5. Phosphates, hydroxides, carbonates, clays, and organic materials can all react with soluble Pb to reduce the solubility of the metal in the soil and, as a result, the amount of Pb that is available to plants is limited [89].

### 4.4. Comparative Levels of Metals with Reported Studies

The PTM levels in the MP and lamina of the present study were comparable to most of the metal levels in the mangrove leaves reported in the literature (Table 14), including those found in nine species of mangroves from Hainan Island, China [1], and in mangrove leaf collected from Punta Mala Bay (Pacific Panama) [5]. The comparatively higher metal levels in the mangrove leaves by those reported by MacFarlane et al. [73], MacFarlane and Burchett [15], and Peng et al. [11] would imply that *Avicennia* could be due to excessive metal deposition and PTM pollution.

According to Kabata-Pendias and Pendias [93], the general PTM concentration for plants was based on the amounts of PTMs that were measured in the plant tissues. The most mobile metal was discovered to be Zn, followed by Cu and Pb in the leaf tissue. The spatial distribution and bio-accumulation of Cu, Ni, Pb, and Zn in marine sediments and *A. marina* at Yanbu Red Sea, Saudi Arabia, were evaluated by Alharbi et al. [94]. They reported that the Cu, Ni, Pb, and Zn concentrations (mg/kg dry weight) in the sediments were 17.2–217.2, 27.3–241.8, 11.5–111.3, and 48.8–511.5, respectively, while the concentrations (mg/kg dry weight) in the leaves were 18.1–40.2, 16.1–56.3, 2.3–9.9, and 36.8–84.9, respectively.

Bakshi et al. [95] reported the significant association between the metal concentration in *A. officinalis* leaves and the sediment metals, and suggests that extensive bioaccumulation had occurred. Chaudhuri et al. [13] reported that *A. marina* accumulated significant amounts of PTMs with a strong positive correlation of metals between the mangrove and sediments (both total and bio-available fractions). Ghasemi et al. [96] reported the phytomanagement of PTMs in mangrove sediments of Hormozgan, Iran, using *A. marina*. In addition, using greenhouse and field tests, Kaewtubtim et al. [97] assessed the uptake and accumulation of PTMs by *Pluchea indica* and *A. marina* and concluded that the mangrove had the potential for phytoremediation.

Using both species-level analyses and a phylogenetic approach, MacFarlane et al. [18] conducted a comparative analysis assessing the patterns of accumulation and partitioning of Cu, Pb and Zn in mangroves from the existing field-based studies to date. *Avicennia* mangroves have several adaptive mechanisms for overcoming the difficulty of saline and extremely anoxic conditions. Although they are different species of mangrove, the metal accumulation and partitioning for Cu, Pb, and Zn were discovered to be comparable across the *Avicennia* genera and vast geographical ecosystems. They concluded that, regardless of ambient quantities, the non-essential metal Pb was not found in the leaf tissue. As a result, mangroves are a species that exclude non-essential metals and regulate vital metals. Mangrove habitats are arguably the best phytostabilizers in terms of their phytoremediation efforts, with the ability to help with the retention of PTMs to reduce transmission to nearby estuarine and marine systems.

Nath et al. [98] reported that the TF values of essential metals such as Cu and Zn were higher than those of non-essential Pb. This implies that *Avicennia* selectively exclude non-essential metals, while regulating essential metals. This could help reduce the PTM toxicity to plants. This also suggests that *A. marina* could act as phytostabilizers that could help the aquatic ecosystem avoid direct or indirect sources of PTM contamination.

### 4.5. The Need for Conservation at Klang Mangrove Ecosystem

The results of the current study supported the fact that there is extensive anthropogenic activity there. This agrees with Naji and Ismail [29], who showed that the highest concentrations of metals were found in stations with high anthropogenic discharge based on the sediments of Klang Estuary. El Turk et al. [17] concluded that future environmental policies towards a sustainable development in the KME require the public awareness to mitigate the pollution problem.

The KME needs further long-term management and conservation measures to be protected. These findings give accurate and reliable data on the accumulation and translocation of PTMs in rhizosphere sediment to plant tissues of *A. apiculata* from the mangrove ecosystem. The study can enhance coastal management and environmental protection initiatives by providing decision-makers with a clear understanding of the existing pollution state of this stressed estuarine environment. The emphasis of the current study was on the potential for developing a framework for managing the KME. In order to mitigate the existing threats to the KME, the information offered in the present study can be employed in the monitoring and detection of PTM pollution in the KME.

The current ecological-health risks of PTMs in the KME are based on an informed and risk-based assessment of the current conditions and potential future scenarios, which can be more likely to be effective and sustainable if the goal is to gather the best information to identify nexus issues. This study can also aid in the ability to evaluate resource availability, current demand, known consequences, development opportunities, and possible climate change implications in the KME. The most significant aspect of the knowledge at hand is risk assessment and climate resilience building, which can benefit the water, energy, and food nexus. Risk evaluations in the industries, mangrove eco-tourism, natural services, and economy resources sectors should take into account the interrelationships between water, energy, and food, as well as the challenges posed by climate change.

Since mangrove forest ecosystem management plans and efforts have been proposed in Malaysia [20,99], the present ecological-health risk of PTM in the KME can be an assert (substantial background information) in formulating the Nexus thinking approach [100] to connect the food-energy-water integration to cater to the ever-growing expansion of new ideas, such as a circular and green economy [101], ‘Good health and well-being’ under the United Nation’s Sustainability Development Goals [102] and One Health concept. There is little question about the need to connect economic, social, and environmental perspectives for the holistic solution using the Nexus approach that was proposed in Cyprus [103].

## 5. Conclusions

This study aimed to evaluate the ecological-health risks of PTMs in the surface sediments collected from the KME, and to assess the phytoremediation potential of *Avicennia officinalis* collected from the KME. The ER values indicated that Ni, Pb and Zn were classified as ‘low potential ecological risk’ (ER < 40)’, while Cu ranged from ‘low potential ecological risk’ to ‘considerable potential ecological risk’. In terms of the health risks of the sediments, all of the HI values of Cu, Ni, Pb and Zn based on the combination of three pathways indicated <1.00, showing that the four metals are non-carcinogenic. Based on the BCF values, it can be concluded that the lamina has a better potential as a phytoremediator of essential Cu, Zn and Fe, while MP has a better potential as a phytoremediator of non-essential Pb and Ni.

Future research should concentrate on tracking the changes in the metal concentrations in sediments and mangrove plants throughout time. It is important to look into the speciation and readily accessible portions of PTMs in sediment. Further research is needed to determine how mangrove systems might reduce PTM contamination using phytoextraction and phytostabilization techniques in terms of the environmental restoration and management of the KME.

## Figures and Tables

**Table 1:** Overall values of concentrations (mg/kg dry weight) for Cu, Fe, Ni, Pb and Zn, of surface sediments in Klang River estuary (8 sites) and a site in Juru estuary (1 site). N = 9.

	Cu Total	Total Fe	Ni Total	Pb Total	Zn Total
Minimum	5.30	22,121	13.90	30.30	46.40
Maximum	63.80	27,906	32.70	62.30	269.00
Mean	38.24	23,735	18.22	46.94	163.60
Median	37.90	22,931	15.90	45.90	168.00
SD	17.29	2167	5.75	9.87	61.39
SE	5.76	885	1.92	3.29	20.46
Skewness	−0.45	1.391	1.98	−0.03	−0.25
Kurtosis	−0.30	0.422	2.73	−0.75	0.16

**Table 2:** Overall values of geoaccumulation index (I_geo_), contamination factor (CF), ecological risk (ER) for Cu, Ni, Pb and Zn, of surface sediments in Klang River estuary and a site in Juru estuary, based on background levels of the metals that were reported from Peninsular Malaysia. N = 9.

	Cu I_geo_	Ni I_geo_	Pb I_geo_	Zn I_geo_	Cu CF	Ni CF	Pb CF	Zn CF	Cu ER	Ni ER	Pb ER	Zn ER
Minimum	−0.01	0.36	0.05	1.23	1.49	1.93	1.55	3.53	7.45	9.64	7.77	3.53
Maximum	3.58	1.60	1.09	3.77	18.0	4.54	3.20	20.5	89.9	22.7	16.0	20.5
Mean	2.60	0.70	0.65	2.92	10.8	2.53	2.41	12.4	53.9	12.6	12.1	12.4
Median	2.83	0.56	0.65	3.09	10.68	2.21	2.35	12.79	53.41	11.05	11.77	12.79
SD	1.07	0.37	0.32	0.72	4.87	0.80	0.51	4.67	24.35	4.00	2.53	4.67
SE	0.36	0.12	0.11	0.24	1.62	0.27	0.17	1.56	8.12	1.33	0.84	1.56
Skewness	−1.77	1.62	−0.44	−1.48	−0.45	1.98	−0.03	−0.24	−0.45	1.98	−0.03	−0.24
Kurtosis	2.19	1.81	−0.40	1.67	−0.29	2.72	−0.75	0.16	−0.29	2.74	−0.75	0.16

Note: The values of I_geo_, CF and ER for Fe were not determined because the toxic response factor for Fe is not available.

**Table 3:** Multiple stepwise regression analytical outputs based on hazard index (HI) and ecological risk (ER) as dependent variables and independent variables are the hazard quotient pathways and geochemical fractions of the sediments.

Cu	Intercept	CF	HI-A						F	df
ER	0.416195	1.130	−0.140						93,254	2.24
Cu	Intercept	HQ_ing_-C	F2						F	df
HI-C	1.53 × 10^−6^	1.000	−0.001						2,661,948	2.24
Cu	Intercept	HQ_inh_-A	I_geo_	CF					F	df
HI-A	−1.8 × 10^−5^	0.988	−0.020	0.030					430,755	3.23
Pb	Intercept	CF	HQ_der_-C	F2					F	df
ER	0.313704	1.340	−0.340	0.004					197,907	3.23
Pb	Intercept	HQ_ing_-C	SUM	F1	F3	F2	I_geo_	F4	F	df
HI-C	1.42 × 10^−3^	1.010	−0.020	0.012	−0.001	−0.010	−0.020	0.034	536,810	7.19
Pb	Intercept	HQ_ing_-A	ER	F1					F	df
HI-A	−5.9 × 10^−4^	0.967	0.033	0.003					680,078	3.23
Zn	Intercept	F1	F2	F3	F4	SUM			F	df
ER	−2.50988	−0.46	−0.64	−0.88	−1.2	4.06			3077	5.21
Zn	Intercept	HQ_ing_-C	F4	F1	I_geo_				F	df
HI-C	−2.6 × 10^−5^	0.999	0.003	0.002	−0.001				16,179,100	4.22
Zn	Intercept	HQ_inh_-A	F1						F	df
HI-A	−7.4 × 10^−6^	0.987	0.013						336,680	2.24
Ni	Intercept	CF	HQ_ing_-C						F	df
ER	0.357376	1.25	−0.26						229,931	2.24
Ni	Intercept	HQ_der_-C	I_geo_						F	df
HI-C	4.47 × 10^−5^	0.98	0.17						1,594,926	2.24
Ni	Intercept	HQ_der_-A							F	df
HI-A	1.6 × 10^−6^	1.00							1,647,769	1.25

Note: The independent variables included the pathways of HQ_ing_, HQ_inh_, and HQ_der_, and ecological indexes (I_geo_, CF, and ER) and geochemical fractions (F1, F2, F3, F4, SUM) of sediments; -C= Children; -A= Adults; All values have been log10 (value+1) prior to multiple linear (forward) stepwise regression analysis; All R values in all equations are 0.999; *p* values in all equations are 0.000; All equations’ N = 27.

**Table 4:** Overall values of hazard quotient (HQ), and hazard index (HI), in the three exposure routes of Ni for children (C) and adults (A) in Klang River estuary and a site in Juru estuary. N = 9.

	HQ_ing_-C	HQ_ing_-A	HQ_inh_-C	HQ_inh_-A	HQ_der_-C	HQ_der_-A	HI-C	HI-A
Minimum	9.11 × 10^−3^	1.22 × 10^−2^	2.42 × 10^−7^	4.36 × 10^−7^	5.40 × 10^−5^	5.51 × 10^−4^	9.16 × 10^−3^	1.77 × 10^−3^
Maximum	2.15 × 10^−2^	2.88 × 10^−2^	5.70 × 10^−7^	1.03 × 10^−6^	1.27 × 10^−5^	1.30 × 10^−3^	2.16 × 10^−2^	4.18 × 10^−3^
Mean	1.19 × 10^−2^	1.60 × 10^−2^	3.17 × 10^−7^	5.72 × 10^−7^	7.08 × 10^−5^	7.23 × 10^−4^	1.20 × 10^−2^	2.33 × 10^−3^
Median	1.04 × 10^−2^	1.40 × 10^−2^	2.78 × 10^−7^	5.00 × 10^−7^	6.19 × 10^−5^	6.32 × 10^−4^	1.05 × 10^−2^	2.03 × 10^−3^
SD	3.80 × 10^−3^	5.08 × 10^−4^	1.00 × 10^−7^	1.82 × 10^−7^	2.23 × 10^−5^	2.29 × 10^−4^	3.81 × 10^−3^	7.37 × 10^−4^
SE	1.27 × 10^−3^	1.69 × 10^−4^	3.35 × 10^−8^	6.06 × 10^−8^	7.45 × 10^−6^	7.64 × 10^−5^	1.27 × 10^−3^	2.46 × 10^−4^
Skewness	1.98	1.98	1.99	1.99	1.98	1.99	1.97	1.98
Kurtosis	2.74	2.74	2.74	2.76	2.73	2.75	2.71	2.73

Note: SD = standard deviation; SE = standard error.

**Table 5:** Overall values of hazard quotient (HQ), and hazard index (HI), in the three exposure routes of Cu for children (C) and adults (A) in in Klang River estuary and a site in Juru estuary. N= 9.

	HQ_ing_-C	HQ_ing_-A	HQ_inh_-C	HQ_inh_-A	HQ_der_-C	HQ_der_-A	HI-C	HI-A
Minimum	1.73 × 10^−3^	2.33 × 10^−4^	4.72 × 10^−8^	2.12 × 10^−8^	9.25 × 10^−6^	2.36 × 10^−5^	1.74 × 10^−3^	2.56 × 10^−4^
Maximum	2.09 × 10^−2^	2.81 × 10^−3^	5.69 × 10^−7^	2.56 × 10^−7^	1.12 × 10^−4^	2.85 × 10^−4^	2.10 × 10^−2^	3.09 × 10^−3^
Mean	1.25 × 10^−2^	1.68 × 10^−3^	3.41 × 10^−7^	1.54 × 10^−7^	6.69 × 10^−5^	1.71 × 10^−4^	1.26 × 10^−2^	1.85 × 10^−3^
Median	1.24 × 10^−2^	1.67 × 10^−3^	3.38 × 10^−7^	1.52 × 10^−7^	6.63 × 10^−5^	1.69 × 10^−4^	1.25 × 10^−2^	1.84 × 10^−3^
SD	5.67 × 10^−3^	7.60 × 10^−4^	1.54 × 10^−7^	6.94 × 10^−8^	3.03 × 10^−5^	7.72 × 10^−5^	5.70 × 10^−3^	8.39 × 10^−4^
SE	1.89 × 10^−3^	2.53 × 10^−4^	5.14 × 10^−8^	2.31 × 10^−8^	1.01 × 10^−5^	2.57 × 10^−5^	1.90 × 10^−3^	2.80 × 10^−4^
Skewness	−0.45	−0.44	−0.45	−0.45	−0.43	−0.45	−0.45	−0.45
Kurtosis	−0.30	−0.29	−0.29	−0.29	−0.29	−0.29	−0.29	−0.31

Note: SD = standard deviation; SE = standard error.

**Table 6:** Overall values of hazard quotient (HQ), and hazard index (HI), in the three exposure routes of Pb for children (C) and adults (A) in in Klang River estuary and a site in Juru estuary. N= 9.

	HQ_ing_-C	HQ_ing_-A	HQ_inh_-C	HQ_inh_-A	HQ_der_-C	HQ_der_-A	HI-C	HI-A
Minimum	1.12 × 10^−1^	1.50 × 10^−2^	3.09 × 10^−6^	1.39 × 10^−6^	1.21 × 10^−3^	3.09 × 10^−3^	1.13 × 10^−1^	1.81 × 10^−2^
Maxim um	2.31 × 10^−1^	3.10 × 10^−2^	6.35 × 10^−6^	2.86 × 10^−6^	2.49 × 10^−3^	6.36 × 10^−3^	2.33 × 10^−1^	3.73 × 10^−2^
Mean	1.74 × 10^−1^	2.33 × 10^−2^	4.78 × 10^−6^	2.15 × 10^−6^	1.87 × 10^−3^	4.79 × 10^−3^	1.76 × 10^−1^	2.81 × 10^−2^
Median	1.70 × 10^−1^	2.28 × 10^−2^	4.67 × 10^−6^	2.10 × 10^−6^	1.83 × 10^−3^	4.68 × 10^−3^	1.72 × 10^−1^	2.75 × 10^−2^
SD	3.65 × 10^−2^	4.93 × 10^−3^	1.00 × 10^−6^	4.53 × 10^−7^	3.95 × 10^−4^	1.01 × 10^−3^	3.69 × 10^−2^	5.91 × 10^−3^
SE	1.22 × 10^−2^	1.64 × 10^−3^	3.35 × 10^−7^	1.51 × 10^−7^	1.32 × 10^−4^	3.36 × 10^−4^	1.23 × 10^−2^	1.97 × 10^−3^
Skewness	−0.03	−0.03	−0.02	−0.02	−0.02	−0.02	−0.04	−0.04
Kurtosis	−0.73	−0.75	−0.75	−0.75	−0.76	−0.75	−0.74	−0.74

Note: SD= standard deviation; SE= standard error.

**Table 7:** Overall values of hazard quotient (HQ), and hazard index (HI), in the three exposure routes of Zn for children (C) and adults (A) in Klang River estuary and a site in Juru estuary. N = 9.

	HQ_ing_-C	HQ_ing_-A	HQ_inh_-C	HQ_inh_-A	HQ_der_-C	HQ_der_-A	HI-C	HI-A
Minimum	2.03 × 10^−3^	2.72 × 10^−4^	5.55 × 10^−8^	2.50 × 10^−8^	1.62 × 10^−5^	4.14 × 10^−5^	2.04 × 10^−3^	3.14 × 10^−4^
Maximum	1.18 × 10^−2^	1.58 × 10^−3^	3.22 × 10^−7^	1.45 × 10^−7^	9.42 × 10^−5^	2.41 × 10^−4^	1.19 × 10^−2^	1.82 × 10^−3^
Mean	7.15 × 10^−3^	9.59 × 10^−4^	1.96 × 10^−7^	8.80 × 10^−8^	5.72 × 10^−5^	1.46 × 10^−4^	7.21 × 10^−3^	1.11 × 10^−3^
Median	7.35 × 10^−3^	9.86 × 10^−4^	2.01 × 10^−7^	9.06 × 10^−8^	5.88 × 10^−5^	1.50 × 10^−4^	7.41 × 10^−3^	1.14 × 10^−3^
SD	2.69 × 10^−3^	3.60 × 10^−4^	7.34 × 10^−8^	3.31 × 10^−8^	2.15 × 10^−5^	5.50 × 10^−5^	2.72 × 10^−3^	4.16 × 10^−4^
SE	8.97 × 10^−4^	1.20 × 10^−4^	2.45 × 10^−8^	1.10 × 10^−8^	7.17 × 10^−6^	1.83 × 10^−5^	9.06 × 10^−4^	1.39 × 10^−4^
Skewness	−0.23	−0.24	−0.25	−0.24	−0.24	−0.24	−0.23	−0.24
Kurtosis	0.17	0.17	0.17	0.17	0.16	0.18	0.16	0.15

Note: SD = standard deviation; SE = standard error.

**Table 8:** Overall statistics of zinc (Zn) concentrations (mg/kg dry weight) in the midrib plus petiole (MP), and lamina of *Avicennia officinalis*, and their bioconcentration factors (BCF) values, collected from 6 sampling sites in the mangrove areas of west coast of Peninsular Malaysia.

MP	Plant	BCF1	BCF2	BCF3	BCF4	BCF5
Minimum	16.4	0.51	0.32	0.37	0.25	0.08
Maximum	36.2	3.02	1.89	1.10	0.91	0.35
Mean	22.9	1.10	0.68	0.53	0.43	0.15
Median	21.2	0.75	0.44	0.43	0.32	0.11
SD	6.79	0.96	0.60	0.28	0.25	0.10
SE	2.77	0.39	0.24	0.12	0.10	0.04
Skewness	1.40	1.63	1.71	1.73	1.35	1.68
Kurtosis	0.66	0.90	1.06	1.10	0.26	1.02
Lamina	Plant	BCF1	BCF2	BCF3	BCF4	BCF5
Minimum	17.57	0.56	0.35	0.34	0.26	0.09
Maximum	41.11	3.65	2.29	1.33	1.10	0.42
Mean	26.92	1.37	0.82	0.62	0.51	0.18
Median	24.05	0.68	0.51	0.46	0.43	0.13
SD	9.91	1.27	0.75	0.38	0.32	0.13
SE	4.05	0.52	0.31	0.15	0.13	0.05
Skewness	0.50	1.13	1.52	1.29	1.18	1.36
Kurtosis	−1.37	−0.34	0.66	0.13	0.08	0.32

Note: BCF1 = leave/F1; BCF2 = leave/F2; BCF3 = leave/F3; BCF4 = leave/F4; BCF5 = leave/SUM.

**Table 9:** Multiple linear stepwise regression analytical outputs based on *Avicennia* leaves (lamina (L); midrib plus petiole (M)) as dependent variables and independent variables are the bioconcentration factors and sedimentary geochemical fractions.

Fe	Intercept	BCF5	BCF2	F4						R	F	df
Lamina	0.64801305	0.621	0.269	0.226						0.995	427	3.14
Fe	Intercept	BCF5	F2	F4	BCF4	SUM				R	F	df
MP	0.99750357	−1.400	−0.210	0.904	1.91	−0.36				0.996	293	5.12
Pb	Intercept	BCF3	BCF1	BCF5	BCF4	F3	SUM	F1	F4	R	F	df
Lamina	0.11027971	−0.040	1.050	−0.120	0.248	−0.19	0.201	0.099	0.088	0.999	4,788,659	8.9
Pb	Intercept	BCF3	F3	F2	F1	BCF5	BCF1	BCF2	SUM	R	F	df
MP	0.365423	−0.260	−0.310	−0.150	0.401	0.112	1.37	−0.32	0.198	0.999	41,252	8.9
Ni	Intercept	BCF3	BCF5	BCF2	BCF4	F2	F1	BCF1		R	F	df
Lamina	−0.10137913	0.577	−1.700	2.770	0.46	1.36	−0.150	−0.300		0.999	34,314	7.10
Ni	Intercept	BCF3	F3	F4	SUM	BCF1	BCF4			R	F	df
MP	0.4677638	1.040	0.827	0.877	−1.60	0.913	−1			0.999	2268	6.11
Cu	Intercept	F1	BCF1	F3	F4					R	F	df
Lamina	0.42926263	1.410	1.960	2.480	−1.80					0.991	186	4.13
Cu	Intercept	F3	BCF4	BCF2	F1	BCF5	F2	BCF1		R	F	df
MP	−0.02309005	0.868	2.310	0.578	−0.07	−1.60	0.418	−0.49		0.999	1019	7.10
Zn	Intercept	BCF4	F4	BCF5	BCF1	F1	BCF2			R	F	df
Lamina	0.70790607	0.620	−1.700	−1.300	5.59	3.41	−2.900			0.997	341	6.11
Zn	Intercept	F3	BCF4	F1						R	F	df
MP	0.11150533	0.999	1.000	0.183						0.982	131	3.23

Note: The independent variables included BCF1, BCF2, BCF3, BCF4, BCF5, and sedimentary geochemical fractions (F1, F1, F3, F4 and SUM). All values have been log_10_ (value + 1) prior to multiple (forward) stepwise regression analysis. All *p* values in all equations are 0.000; All equations’ N = 18.

**Table 10:** Overall statistics of iron (Fe) concentrations (mg/kg dry weight) in the midrib plus petiole (MP), and lamina of *Avicennia officinalis*, and their bioconcentration factors (BCF) values, collected from 6 sampling sites in the mangrove areas of west coast of Peninsular Malaysia.

MP	Plant	BCF1	BCF2	BCF3	BCF4	BCF5
Minimum	45.8	0.170	0.020	0.004	0.003	0.002
Maximum	193	0.970	0.240	0.098	0.008	0.007
Mean	117	0.463	0.117	0.050	0.005	0.004
Median	118	0.340	0.100	0.049	0.005	0.004
SD	57.5	0.314	0.087	0.035	0.002	0.002
SE	23.5	0.128	0.036	0.014	0.001	0.001
Skewness	0.036	0.721	0.362	0.059	0.466	0.287
Kurtosis	−1.481	−1.000	−1.325	−1.277	−1.283	−1.180
Lamina	Plant	BCF1	BCF2	BCF3	BCF4	BCF5
Minimum	92.9	0.340	0.040	0.009	0.006	0.004
Maximum	244	1.230	0.300	0.142	0.010	0.009
Mean	166	0.647	0.163	0.073	0.007	0.006
Median	172	0.495	0.160	0.072	0.007	0.006
SD	53.6	0.340	0.105	0.048	0.002	0.002
SE	21.9	0.139	0.043	0.020	0.001	0.001
Skewness	0.01	0.923	0.088	0.126	0.927	0.689
Kurtosis	−0.96	−0.659	−1.568	−1.160	−0.332	−0.656

Note: BCF1 = leave/F1; BCF2 = leave/F2; BCF3 = leave/F3; BCF4 = leave/F4; BCF5 = leave/SUM.

**Table 11:** Overall statistics of lead (Pb) concentrations (mg/kg dry weight) in the midrib plus petiole (MP), and lamina of *Avicennia officinalis*, and their bioconcentration factors (BCF) values, collected from 6 sampling sites in the mangrove areas of west coast of Peninsular Malaysia.

MP	Plant	BCF1	BCF2	BCF3	BCF4	BCF5
Minimum	6.17	6.93	10.64	0.34	0.19	0.12
Maximum	23.67	22.76	29.96	1.23	4.90	0.62
Mean	11.91	12.78	15.78	0.62	1.26	0.32
Median	10.54	12.75	13.70	0.52	0.55	0.23
SD	6.70	5.49	7.10	0.36	1.82	0.22
SE	2.74	2.24	2.90	0.15	0.74	0.09
Skewness	0.88	0.96	1.61	0.78	1.65	0.60
Kurtosis	−0.45	0.00	0.90	−0.70	0.95	−1.43
Lamina	Plant	BCF1	BCF2	BCF3	BCF4	BCF5
Minimum	3.39	3.81	5.84	0.18	0.10	0.07
Maximum	20.58	19.98	22.57	1.23	7.62	0.96
Mean	10.92	11.4	13.5	0.58	1.61	0.32
Median	10.16	11.56	11.99	0.48	0.48	0.21
SD	7.49	6.51	6.92	0.44	2.96	0.34
SE	3.06	2.66	2.83	0.18	1.21	0.14
Skewness	0.17	0.03	0.34	0.45	1.75	1.28
Kurtosis	−1.64	−1.41	−1.45	−1.33	1.14	0.22

Note: BCF1 = leave/F1; BCF2 = leave/F2; BCF3 = leave/F3; BCF4 = leave/F4; BCF5 = leave/SUM.

**Table 12:** Overall statistics of copper (Cu) concentrations (mg/kg dry weight) in the midrib plus petiole (MP), and lamina of *Avicennia officinalis*, and their bioconcentration factors (BCF) values, collected from 6 sampling sites in the mangrove areas of west coast of Peninsular Malaysia.

MP	Plant	BCF1	BCF2	BCF3	BCF4	BCF5
Minimum	3.77	2.78	66.1	0.39	0.12	0.09
Maximum	11.6	15.7	601	377	0.53	0.51
Mean	6.88	7.33	317	63.4	0.26	0.21
Median	6.31	6.30	333	0.72	0.20	0.16
SD	2.61	4.44	183	154	0.15	0.15
SE	1.06	1.81	74.6	62.7	0.06	0.06
Skewness	0.87	1.20	0.20	1.79	1.12	1.50
Kurtosis	−0.02	0.33	−0.69	1.20	−0.07	0.68
Lamina	Plant	BCF1	BCF2	BCF3	BCF4	BCF5
Minimum	5.56	2.81	66.8	0.40	0.12	0.09
Maximum	11.9	31.1	746	746	1.04	1.00
Mean	7.76	10.1	381	125	0.35	0.30
Median	7.43	7.13	335.00	0.74	0.22	0.17
SD	2.20	10.43	239.48	304.28	0.34	0.35
SE	0.90	4.26	97.77	124.22	0.14	0.14
Skewness	1.31	1.67	0.32	1.79	1.63	1.72
Kurtosis	0.53	1.01	−0.90	1.20	0.90	1.07

Note: BCF1 = leave/F1; BCF2 = leave/F2; BCF3 = leave/F3; BCF4 = leave/F4; BCF5 = leave/SUM.

**Table 13:** Overall statistics of nickel (Ni) concentrations (mg/kg dry weight) in the midrib plus petiole (MP), and lamina of *Avicennia officinalis*, and their bioconcentration factors (BCF) values, collected from 6 sampling sites in the mangrove areas of the west coast of Peninsular Malaysia.

MP	Plant	BCF1	BCF2	BCF3	BCF4	BCF5
Minimum	0.21	0.09	0.06	0.01	0.01	0.01
Maximum	4.09	12.78	4.49	0.64	0.66	0.30
Mean	2.07	3.59	1.70	0.32	0.25	0.13
Median	1.99	2.16	1.10	0.33	0.21	0.11
SD	1.37	4.61	1.58	0.22	0.22	0.10
SE	0.56	1.88	0.65	0.09	0.09	0.04
Skewness	0.16	1.60	0.94	0.04	1.02	0.84
Kurtosis	−0.93	0.88	−0.35	−0.83	0.09	−0.19
Lamina	Plant	BCF1	BCF2	BCF3	BCF4	BCF5
Minimum	0.13	0.05	0.04	0.01	0.01	0.00
Maximum	2.86	8.94	3.14	0.45	0.46	0.21
Mean	1.74	2.84	1.41	0.27	0.21	0.10
Median	1.86	2.11	1.23	0.28	0.21	0.10
SD	0.93	3.10	1.07	0.15	0.15	0.07
SE	0.38	1.27	0.44	0.06	0.06	0.03
Skewness	−0.69	1.48	0.45	−0.73	0.51	0.15
Kurtosis	−0.36	0.74	−0.70	−0.07	−0.15	−0.24

Note: BCF1 = leave/F1; BCF2 = leave/F2; BCF3 = leave/F3; BCF4 = leave/F4; BCF5 = leave/SUM.

**Table 14:** Comparison of concentrations (mg/kg dry weight) of Zn, Pb, and Cu in the leaves of *Avicennia officinalis* and their bioconcentration factor (BCF) values.

Location		Zn	Zn BCF	Pb	Pb BCF	Cu	Cu BCF	Reference
Klang and Juru mangrove	MP	16.4–36.2	0.08–0.35	6.17–23.7	0.12–0.62	3.77–11.6	0.09–0.51	This study
	Lamina	17.6–41.1	0.09–0.42	3.39–20.6	0.07–0.96	5.56–11.9	0.09–1.00	
	SED	46.4–269	-	30.3–62.2	-	5.29–63.8	-	
East India	Leaf	1.20	0.80	NA	-	2.9	0.46	Sarangi et al. [90]
	SED	1.50	-	NA	-	6.3	-	
India	Leaf	120	0.16	168	0.11	102	0.34	Thomas and Fernandez [91]
	SED	764	-	1484	-	303	-	
India	Leaf	120	-	168	-	102	-	Tam and Wong [92]
India	Leaf	18.5–28.3	-	3.26–4.57	-	6.76–11.9	-	Chakraborty et al. [56]
	SED	56.4–61.5	-	28.8–36.8	-	34.3–40.7	-	

Note: SED = sediment; NA = not available.

## Data Availability

Not applicable.

## Supplementary Materials

The following supporting information can be downloaded at: https://www.mdpi.com/article/10.3390/biology12010043/s1, Table S1: Sampling information in the mangrove of Klang River (K1-K8) and Juru estuary (J1). Samplings were conducted on 2 December 2007 while Juru estuary on 8 December 2007, Table S2: Heavy metals analysis recovery percentages of the certified reference materials (CRM), Table S3: Comparisons of concentrations (mg/kg dry weight) of Zn, Cu and Zn between surface sediments from this study with those cited from sediment quality guidelines, and reference values, Table S4: Definition, exposure factors, and reference values were used to estimate the intake values and health risks of potentially toxic metals in sediments used in the present study, Table S5: Concentrations (mg/kg dry weight) of Cu, Ni, Fe, Pb and Zn in the geochemical fractions of surface sediments in Klang River estuary (S1–S8) and a site in Juru estuary (S9), Table S6: Values of geoaccumulation index (I_geo_), contamination factor (CF), ecological risk (ER) for Cu, Ni, Pb and Zn, and potentially ecological risk index (PERI) of surface sediments in Klang River estuary (S1–S8) and a site in Juru estuary (S9), based on background levels of the metals that were reported from Peninsular Malaysia, Table S7: Values of hazard quotient (HQ), and hazard index (HI), in the three exposure routes of Ni in Klang River estuary (S1–S8) and a site in Juru estuary (S9), Table S8: Values of hazard quotient (HQ), and hazard index (HI), in the three exposure routes of Cu in in Klang River estuary (S1–S8) and a site in Juru estuary (S9), Table S9: Values of hazard quotient (HQ), and hazard index (HI), in the three exposure routes of Pb in in Klang River estuary (S1–S8) and a site in Juru estuary (S9), Table S10: Values of hazard quotient (HQ), and hazard index (HI), in the three exposure routes of Zn in Klang River estuary (S1–S8) and a site in Juru estuary (S9), Table S11: Mean concentrations (mg/kg dry weight) of Cu, Fe and Pb in the leaf parts (lamina (L), midrib plus petiole (M + P)) of *Avicennia officinalis* and total concentrations of their habitat surface sediments in in the mangrove of Klang estuary (S1, S2, S5, S6 and S8) and Juru estuary (S9) [34,40,41,42,43,44,46,48,49,50,54,67,68,69,70,104,105,106,107,108].
