# Supplementary material for: The Ecological-Health Risks of Potentially Toxic Metals in the Surface Sediments and Leaves of Salt-Secreting Avicennia officinalis as Potential Phytoremediators: A Field-Based Biomonitoring Study from Klang Mangrove Area
Source: Biology (Basel). 2022 Dec 26;12(1):43. doi: 10.3390/biology12010043 (PMC9855768; doi:10.3390/biology12010043)
Supplement: Supplementary file 1 [file biology-12-00043-s001.zip › biology-2090874-supplementary.pdf]

Article

The ecological-health risks of potentially toxic metals in the surface sediments and leaves of salt-secreting *Avicennia officinalis* as potential phytoremediators: A field-based biomonitoring study from Klang mangrove area

Chee Kong Yap [1,*] and Khalid Awadh Al-Mutairi [2]

[1] Department of Biology, Faculty of Science, Universiti Putra Malaysia, Serdang 43400, Malaysia
[2] Department of Biology, Faculty of Science, University of Tabuk, Tabuk P.O. Box 741, Saudi Arabia
* Correspondence: author: yapckong@hotmail.com or yapchee@upm.edu.my

Table S1: Sampling information in the mangrove of Klang River (S1-S8) and Juru estuary (S9). Samplings were conducted on 2 December 2007 while Juru estuary on 8 December 2007.

Site	Latitude	Longitude	Description of sampling sites	Temperature (°C)	Conductivity (µs/cm)	Salinity (ppt)	TDS (g/L)	Dissolve O_2 (mg/L)
S1*	E 101º22.511′	N 03º01.343′,	Mangrove area, 100m away from bridge, domestic waste floating in water.	27.62	14219	7.78	8.801	1.21
S2*	E 101º22.680′	N 03º01.546′,	Mangrove area, stagment water, domestic waste floating in water, behind mosque.	27.75	14399	7.86	8.892	0.5
S3	E 101º23.046′	N 03º01.599′,	Mangrove area, 600m away from bridge, the beavers around here.	27.67	13769	7.51	8.515	0.07
S4	E 101º20.560′	N 03º01.044′,	Mangrove area, 1000m away from bridge, factory beside the river.	28.03	6279	3.2	3.858	0.02
S5*	E 101º20.751′	N 03º01.796′,	Mangrove area and dirty water.	28.67	5310	2.64	3.226	0.08
S6*	E 101º21.150′	N 03º01.615′,	Mangrove area, dirty water.	27.03	6612	3.46	4.137	0.08
S7	E 101º22.720′	N 03º01.468′,	Mangrove area, water not so dirty, 400m from water tank.	28.85	13246	7.02	8.02	0.01
S8*	E 101º23.098′	N 02º59.570′,	Estuary area, 800m away from industry area.	29.09	26717	14.99	16.11	-0.19
S9*	E 100°373068	N5°350958′	Mangrove area besides the jetty, low tide, opposite to Pulau Pinang industrial area, muddy area, near the fishing village area, boat activities around here	NA	NA	NA	NA	NA

Note: * indicates samplings sites with mangrove leaves collected.

Table S2. Heavy metals analysis recovery percentages of the certified reference materials (CRM).

CRM	Cu	Fe	Ni	Pb	Zn
NSC DC73319 Soil China	85.0%	NA	NA	99.8%	99.7%
MESS - 3 NRC	93.1%	NA	102%	116%	82.8%
TH-1 Sediment Canada	92.9%	95.6%	112%	100%	110%
SRM 1547	NA	106%	NA	NA	115%
IAEA Soil-5	91.3%	NA	103%	116%	94.8%

NA - data not available.

Table S3. Comparisons of concentrations (mg/kg dry weight) of Zn, Cu and Zn between surface sediments from this study with those cited from sediment quality guidelines, and reference values.

	Zn	Cu	Fe	Ni	Pb	References
**Reference values**			**-**			
Pre-industrial reference level	175	50.0	-	NA	70.0	Hakanson [1]
Upper continental crust	52.0	14.3	43000	19.0	17.0	Wedepohl [2]
Upper continental crust	71.0	25.0	-	44.0	17.0	Taylor and McLennan [3]
Upper continental crust	67.0	28.0	-	47.0	17.0	Rudnick and Gao [4]
Background of WCPM	-	3.55	-		-	Yap *et al.* [5]
Background of WCPM	-	-	-	7.31	-	Yap et al. [6]
Background of WCPM	-	-	-	-	19.48	Yap and Noorhaidah [7]
Background of WCPM	13.16	-	-	-	-	Yap *et al.* [8]
Mangrove of west coast of PM	29.4-130	5.59-28.7	-	-	25.4-173	Cheng and Yap [9]
Mangrove of west coast of PM	-	-	-	4.33-24.1	-	Cheng et al. [10]
Klang and Juru mangrove area	46.4-269	5.29-63.8	22121-27906	14.2-32.7	30.3-62.2	This study
**Sediment Quality Guidelines**						
Effect range low (ERL)	150	34.0	-	20.9	46.7	Long et al. [11]
Effects range median (ERM)	410	270	-	51.6	218	Long et al. [11]
Threshold effect level (TEL)	124	18.7	-	15.9	30.2	Macdonald et al. [12]
Probable effect level (PEL)	271	108	-	42.8	112	Macdonald et al. [12]
Interim sediment quality value (ISQV)-low	200	65.0	-	40.0	75.0	Chapman et al. [13]
Interim sediment quality value (ISQV)-high	410	270	-	NA	218	Chapman et al. [13]

Table S4. Definition, exposure factors, and reference values were used to estimate the intake values and health risks of potentially toxic metals in sediments used in the present study.

	Definition	Unit	Values		References
			Children	Adults	
IngR	Ingestion rate of sediment	mg/day	200	100	USEPA [14]
InhR	Inhalation rate of sediment	m^3/day	7.63	12.8	Li et al. [15]
BW	Body weight of the exposed individual	kg	15.0	55.9	BQTSB [16]
EF	Exposure frequency	days/year	350	350	BQTSB [16]
ED	Exposure duration	years	6	24	USEPA [14]
AT	Average time	days	365 × ED	365 × ED	USEPA [17]
PEF	Particle emission factor	m^3/kg	1.36×10^9	1.36×10^9	USEPA [14]
SA	Exposed skin surface area	cm^2	1600	4350	BQTSB [16]
AF	Skin adherence factor	mg/cm day	0.20	0.70	Barnes et al. [18]
ABF	Dermal absorption factor	unitless	0.001	0.001	Chabukdhara and Nema [19]
Cu RfD	Reference dose for ingestion	mg/kg day	4.00×10^{-2}	4.00×10^{-2}	Qing et al. [20]
Cu RfD	Reference dose for inhalation	mg/kg day	4.02×10^{-2}	4.02×10^{-2}	Qing et al. [20]
Cu RfD	Reference dose for dermal contact	mg/kg day	1.20×10^{-2}	1.20×10^{-2}	Qing et al. [20]
Ni RfD	Reference dose for ingestion	mg/kg day	2.00E−02	2.00E−02	Qing et al. [20]
Ni RfD	Reference dose for inhalation	mg/kg day	2.06E−02	2.06E−02	Qing et al. [20]
Ni RfD	Reference dose for dermal contact	mg/kg day	5.40E−03	5.40E−03	Qing et al. [20]
Pb RfD	Reference dose for ingestion	mg/kg day	3.50×10^{-3}	3.50×10^{-3}	Qing et al. [20]
Pb RfD	Reference dose for inhalation	mg/kg day	3.52×10^{-3}	3.52×10^{-3}	Qing et al. [20]
Pb RfD	Reference dose for dermal contact	mg/kg day	5.25×10^{-4}	5.25×10^{-4}	Qing et al. [20]
Zn RfD	Reference dose for ingestion	mg/kg day	3.00×10^{-1}	3.00×10^{-1}	Qing et al. [20]
Zn RfD	Reference dose for inhalation	mg/kg day	3.00×10^{-1}	3.00×10^{-1}	Qing et al. [20]
Zn RfD	Reference dose for dermal contact	mg/kg day	6.00×10^{-2}	6.00×10^{-2}	Qing et al. [20]

Table S5: Concentrations (mg/kg dry weight) of Cu, Ni, Fe, Pb and Zn in the geochemical fractions of surface sediments in Klang River estuary (S1-S8) and a site in Juru estuary (S9).

	Ni					Cu				
Sites	F1	F2	F3	F4	SUM	F1	F2	F3	F4	SUM
S1	0.99	1.20	6.76	10.12	19.08	0.78	0.01	7.65	32.03	40.49
S2	0.85	1.23	6.75	8.39	17.23	1.01	0.03	10.07	31.43	42.54
S3	0.63	1.01	5.41	9.29	16.35	0.59	0.00	7.29	25.61	33.49
S4	0.72	1.46	7.84	9.23	19.26	1.12	0.03	12.44	36.33	49.91
S5	0.92	1.61	5.76	9.02	17.32	1.46	0.02	10.75	35.49	47.72
S6	0.92	2.05	6.48	10.09	19.55	1.68	0.04	15.41	35.90	53.04
S7	0.60	0.85	4.13	7.93	13.51	0.91	0.01	5.76	19.44	26.12
S8	0.32	0.91	6.41	6.19	13.83	0.24	0.01	0.01	7.17	7.43
S9	2.44	3.42	19.72	14.87	40.46	2.38	0.10	16.89	55.22	74.59
	Pb					Zn				
Sites	F1	F2	F3	F4	SUM	F1	F2	F3	F4	SUM
S1	0.89	0.58	17.62	32.69	51.79	19.49	46.43	54.05	72.69	192.66
S2	1.04	0.79	19.19	21.34	42.36	27.24	49.31	52.11	66.50	195.17
S3	0.45	0.44	15.50	39.10	55.49	17.80	39.95	40.18	57.89	155.82
S4	0.63	0.13	23.11	38.43	62.31	26.83	51.89	57.94	73.68	210.34
S5	0.74	0.46	18.57	24.31	44.08	31.91	50.67	44.35	66.44	193.36
S6	1.10	1.19	20.64	36.48	59.41	41.09	54.70	47.98	70.70	214.47
S7	0.13	0.96	12.44	25.56	39.08	13.33	37.04	34.26	51.31	135.94
S8	1.03	0.98	16.75	2.70	21.45	6.86	10.93	18.79	22.82	59.40
S9	0.62	0.53	22.83	11.12	35.09	51.84	61.88	84.98	71.34	270.04
	Fe									
Sites	F1	F2	F3	F4	SUM					
S1	199	974	2659	24542	28374					
S2	260	1783	2408	21222	25673					
S3	125	1852	2263	21074	25314					
S4	232	3065	2470	23784	29551					
S5	380	1533	1642	22828	26383					
S6	376	922	1378	23549	26225					
S7	285	851	1414	19299	21848					
S8	272	2174	10844	12823	26112					
S9	202	600	4581	29152	34535					

Note: F1= easily, freely, leachable or exchangeable fraction; F2= acid-reducible fraction; F3= oxidisable-organic fraction; F4= resistant fraction; SUM= summation of F1, F2, F3 and F4.

Table S6. Values of geoaccumulation index (I_{geo}), contamination factor (CF), ecological risk (ER) for Cu, Ni, Pb and Zn, and potentially ecological risk index (PERI) of surface sediments in Klang River estuary (S1-S8) and a site in Juru estuary (S9), based on background levels of the metals that were reported from Peninsular Malaysia.

Site	Cu I_{geo}	Ni I_{geo}	Pb I_{geo}	Zn I_{geo}	Cu CF	Ni CF	Pb CF	Zn CF	Cu ER	Ni ER	Pb ER	Zn ER
S1	2.82	0.54	0.65	3.01	10.58	2.18	2.35	12.07	52.89	10.92	11.77	12.07
S2	2.83	0.56	0.73	3.09	10.68	2.21	2.49	12.79	53.41	11.05	12.47	12.79
S3	2.44	0.52	0.55	2.75	8.11	2.14	2.20	10.09	40.56	10.72	11.01	10.09
S4	3.06	0.77	0.89	3.21	12.48	2.56	2.78	13.90	62.41	12.80	13.88	13.90
S5	3.17	0.73	0.97	3.22	13.48	2.50	2.94	13.97	67.39	12.48	14.70	13.97
S6	3.34	0.85	1.09	3.39	15.21	2.71	3.20	15.71	76.04	13.53	16.00	15.71
S7	2.21	0.36	0.53	2.64	6.94	1.93	2.17	9.32	34.70	9.64	10.85	9.32
S8	-0.01	0.40	0.05	1.23	1.49	1.97	1.55	3.53	7.45	9.87	7.77	3.53
S9	3.58	1.60	0.41	3.77	17.97	4.54	1.99	20.47	89.87	22.70	9.97	20.47

Table S7. Values of hazard quotient (HQ), and hazard index (HI), in the three exposure routes of Ni in Klang River estuary (S1-S8) and a site in Juru estuary (S9).

		HQ_{ing}		HQ_{inh}		HQ_{der}		Children	Adults
Site	Ni Total	Children	Adults	Children	Adults	Children	Adults	HI	HI
S1	15.8	1.03E-02	1.39E-03	2.74E-07	4.94E-07	6.12E-05	6.25E-04	1.04E-02	2.01E-03
S2	15.9	1.04E-02	1.40E-03	2.78E-07	5.00E-07	6.19E-05	6.32E-04	1.05E-02	2.03E-03
S3	15.5	1.01E-02	1.36E-03	2.69E-07	4.85E-07	6.00E-05	6.13E-04	1.02E-02	1.97E-03
S4	18.5	1.21E-02	1.62E-03	3.21E-07	5.79E-07	7.17E-05	7.32E-04	1.22E-02	2.36E-03
S5	18.0	1.18E-02	1.58E-03	3.13E-07	5.64E-07	6.99E-05	7.14E-04	1.19E-02	2.30E-03
S6	19.5	1.28E-02	1.72E-03	3.40E-07	6.12E-07	7.58E-05	7.74E-04	1.29E-02	2.49E-03
S7	13.9	9.11E-03	1.22E-03	2.42E-07	4.36E-07	5.40E-05	5.51E-04	9.16E-03	1.77E-03
S8	14.2	9.33E-03	1.25E-03	2.48E-07	4.46E-07	5.53E-05	5.65E-04	9.38E-03	1.82E-03
S9	32.7	2.15E-02	2.88E-03	5.70E-07	1.03E-06	1.27E-04	1.30E-03	2.16E-02	4.18E-03

Note: Ni Total= Total Ni concentrations in the surface sediments.

Table S8. Values of hazard quotient (HQ), and hazard index (HI), in the three exposure routes of Cu in in Klang River estuary (S1-S8) and a site in Juru estuary (S9).

		HQ_{ing}		HQ_{inh}		HQ_{der}		Children	Adults
Site	Cu Total	Children	Adults	Children	Adults	Children	Adults	HI	HI
S1	37.6	1.23E-02	1.65E-03	3.35E-07	1.51E-07	6.56E-05	1.68E-04	1.24E-02	1.82E-03
S2	37.9	1.24E-02	1.67E-03	3.38E-07	1.52E-07	6.63E-05	1.69E-04	1.25E-02	1.84E-03
S3	28.8	9.44E-03	1.27E-03	2.57E-07	1.16E-07	5.03E-05	1.29E-04	9.49E-03	1.39E-03
S4	44.3	1.45E-02	1.95E-03	3.95E-07	1.78E-07	7.74E-05	1.98E-04	1.46E-02	2.15E-03
S5	47.9	1.57E-02	2.10E-03	4.27E-07	1.92E-07	8.36E-05	2.14E-04	1.58E-02	2.32E-03
S6	54.0	1.77E-02	2.37E-03	4.82E-07	2.17E-07	9.44E-05	2.41E-04	1.78E-02	2.62E-03
S7	24.6	8.07E-03	1.08E-03	2.20E-07	9.90E-08	4.31E-05	1.10E-04	8.12E-03	1.19E-03
S8	5.30	1.73E-03	2.33E-04	4.72E-08	2.12E-08	9.25E-06	2.36E-05	1.74E-03	2.56E-04
S9	63.8	2.09E-02	2.81E-03	5.69E-07	2.56E-07	1.12E-04	2.85E-04	2.10E-02	3.09E-03

Note: Cu Total= Total Cu concentrations in the surface sediments.

Table S9. Values of hazard quotient (HQ), and hazard index (HI), in the three exposure routes of Pb in in Klang River estuary (S1-S8) and a site in Juru estuary (S9).

		HQ_{ing}		HQ_{inh}		HQ_{der}		Children	Adults
Site	Pb Total	Children	Adults	Children	Adults	Children	Adults	HI	HI
S1	45.9	1.70E-01	2.28E-02	4.67E-06	2.10E-06	1.83E-03	4.68E-03	1.72E-01	2.75E-02
S2	48.6	1.80E-01	2.41E-02	4.95E-06	2.23E-06	1.94E-03	4.95E-03	1.82E-01	2.91E-02
S3	42.9	1.59E-01	2.13E-02	4.37E-06	1.97E-06	1.71E-03	4.37E-03	1.61E-01	2.57E-02
S4	54.1	2.00E-01	2.69E-02	5.51E-06	2.48E-06	2.16E-03	5.52E-03	2.02E-01	3.24E-02
S5	57.3	2.12E-01	2.85E-02	5.84E-06	2.63E-06	2.29E-03	5.84E-03	2.14E-01	3.43E-02
S6	62.3	2.31E-01	3.10E-02	6.35E-06	2.86E-06	2.49E-03	6.36E-03	2.33E-01	3.73E-02
S7	42.3	1.57E-01	2.10E-02	4.31E-06	1.94E-06	1.69E-03	4.31E-03	1.58E-01	2.53E-02
S8	30.3	1.12E-01	1.50E-02	3.09E-06	1.39E-06	1.21E-03	3.09E-03	1.13E-01	1.81E-02
S9	38.8	1.44E-01	1.93E-02	3.96E-06	1.78E-06	1.55E-03	3.96E-03	1.45E-01	2.33E-02

Note: Pb Total= Total Pb concentrations in the surface sediments.

Table S10. Values of hazard quotient (HQ), and hazard index (HI), in the three exposure routes of Zn in Klang River estuary (S1-S8) and a site in Juru estuary (S9).

		HQ_{ing}		HQ_{inh}		HQ_{der}		Children	Adults
Site	Zn Total	Children	Adults	Children	Adults	Children	Adults	HI	HI
S1	159	6.94E-03	9.32E-04	1.90E-07	8.55E-08	5.55E-05	1.42E-04	7.00E-03	1.07E-03
S2	168	7.35E-03	9.86E-04	2.01E-07	9.06E-08	5.88E-05	1.50E-04	7.41E-03	1.14E-03
S3	133	5.80E-03	7.79E-04	1.59E-07	7.15E-08	4.64E-05	1.19E-04	5.85E-03	8.97E-04
S4	183	7.99E-03	1.07E-03	2.19E-07	9.85E-08	6.39E-05	1.63E-04	8.06E-03	1.24E-03
S5	184	8.03E-03	1.08E-03	2.20E-07	9.89E-08	6.43E-05	1.64E-04	8.10E-03	1.24E-03
S6	207	9.03E-03	1.21E-03	2.47E-07	1.11E-07	7.23E-05	1.85E-04	9.11E-03	1.40E-03
S7	123	5.36E-03	7.19E-04	1.47E-07	6.60E-08	4.29E-05	1.10E-04	5.40E-03	8.29E-04
S8	46.4	2.03E-03	2.72E-04	5.55E-08	2.50E-08	1.62E-05	4.14E-05	2.04E-03	3.14E-04
S9	269	1.18E-02	1.58E-03	3.22E-07	1.45E-07	9.42E-05	2.41E-04	1.19E-02	1.82E-03

Note: Zn Total= Total Zn concentrations in the surface sediments.

Table S11: Mean concentrations (mg/kg dry weight) of Cu, Fe and Pb in the leaf parts (lamina (L), midrib plus petiole (M+P)) of *Avicennia officinalis* and total concentrations of their habitat surface sediments (SED) in the mangrove of Klang estuary (S1, S2, S5, S6 and S8) and Juru estuary (S9).

Sites	Parts	Cu Leaves	Cu SED	Fe Leaves	Fe SED	Pb Leaves	Pb SED	Ni Leaves	Ni SED	Zn Leaves	Zn SED
S1	L	5.56	37.55	244.02	24175	3.39	45.8 6	2.33	15.75	41.11	158.90
	M+P	6.01	37.55	192.71	24175	6.17	45.86	3.02	15.75	20.5	158.90
S2	L	7.47	37.92	121.81	22121	17.83	48.57	1.94	15.94	17.57	168.27
	M+P	5.77	37.92	66.68	22121	23.67	48.57	1.12	15.94	21.65	168.27
S5	L	7.41	47.85	167.47	22343	3.4	57.28	1.38	18.00	17.79	183.82
	M+P	7.54	47.85	160.39	22343	6.4	57.28	1.94	18.00	16.41	183.82
S6	L	11.98	53.99	195.26	23095	13.71	62.34	1.78	19.51	23.08	206.77
	M+P	11.55	53.99	92.45	23095	14.14	62.34	2.03	19.51	22.23	206.77
S8	L	7.46	5.29	92.89	22767	20.578	30.29	2.86	14.23	25.02	46.42
	M+P	3.77	5.29	45.83	22767	13.22	30.29	4.09	14.23	20.7	46.42
S9	L	6.68	63.81	177.3	27906	6.6	38.83	0.13	32.74	36.94	269.45
	M+P	6.61	63.81	144.18	27906	7.85	38.83	0.21	32.74	36.15	269.45

References

[1] Hakanson, L. An Ecological Risk Index for Aquatic Pollution Control.a Sedimentological Approach. *Water Res.* **1980**, *14*, 975–1001, doi:10.1016/0043-1354(80)90143-8.

[2] Wedepohl, K.H. The Composition of the Continental Crust. *Geochim. Cosmochim. Acta* **1995**, *59*, 1217–1232, doi:10.1016/0016-7037(95)00038-2.

[3] Taylor, S.R.; McLennan, S.M. The Geochemical Evolution of the Continental Crust. *Rev. Geophys.* **1995**, *33*, 241–265, doi:https://doi.org/10.1029/95RG00262.

[4] Rudnick, R.L.; Gao, S. 3.01 - Composition of the Continental Crust. In *Treatise on Geochemistry*; Holland, H.D., Turekian, K.K., Eds.; Pergamon: Oxford, 2003; 1–64 ISBN 978-0-08-043751-4.

[5] Yap, C.K.; Arifin, N.; Tan, S.G. Relationships of Copper Concentrations between the Different Soft Tissues of *Telescopium telescopium* and the Surface Sediments Collected from Tropical Intertidal Areas. *Int. J. Chem.* **2013**, *5*, 8–19, doi:10.5539/ijc.v5n1p8.

[6] Yap, C.K.; Noorhaidah, A.; Tan, S.G. Digestive Cecum and Tissue Redistribution in Gills of *Telescopium telescopium* as Indicators of Ni Bioavailabilities and Contamination in Tropical Intertidal Areas. *Water. Air. Soil Pollut.* **2012**, *223*, 2891–2905. https://doi.org/10.1007/s11270-012-1073-0.

[7] Yap, C.K.; Noorhaidah, A. Gill and Digestive Caecum of *Telescopium telescopium* as Biomonitors of Pb Bioavailability and Contamination by Pb in the Tropical Intertidal Area. *Sains Malays.* **2011**, *40*, 175–1085.

[8] Yap, C.K.; Noorhaidah, A.; Tan, S.G. Zn Concentrations in the Different Soft Tissues of Telescopium Telescopium and Their Relationships with Zn Speciation by Sequential Extraction in Surface Sediments: A Statistical Multiple Linear Stepwise Regression Analysis. In *Gastropods: Diversity, Habitat, and Genetics*; Branchi, A.M., Fields, J.N., Eds.; Nova Science Publishers Inc.: New York, NY, USA, **2011**; pp. 127–148 ISBN 978-1-61324-695-5.

[9] Cheng, W.H.; Yap, C.K. Potential human health risks from toxic metals via mangrove snail consumption and their ecological risk assessments in the habitat sediment from Peninsular Malaysia. *Chemosphere*, **2015**, *135*, 156–165.

[10] Cheng, W.H., Yap, C.K., Ismail, A. and Abdul Rahim, I. Distribution and concentrations of Ni in tissues of the gastropod Nerita lineata collected from intertidal areas of Peninsular Malaysia. *Pertanika J. Trop. Agric. Sci.*, 2012, 35 (4): 723-736.

[11] Long, E.R.; MacDonald, D.D.; Smith, S.L.; Calder, F.D. Incidence of adverse biological effects within ranges of chemical concentrations in marine and estuarine sediments. *Environ. Manag.*, **1995**, *19*, 81–97.

[12] Macdonald, D.D.; Carr, R.S.; Calder, F.D.; Long, E.R.; Ingersoll, C.G. Development and evaluation of sediment quality guidelines for Florida coastal waters. *Ecotoxicology*, **1996**, *5(4)*, 253–278. https://doi.org/10.1007/bf00118995.

[13] Chapman, P.M.; Allard, P.J.; Vigers, G.A. Development of Sediment Quality Values for Hong Kong Special Administrative Region: A Possible Model for Other Jurisdictions. *Mar. Pollut. Bull.* **1999**, *38*, 161–169, doi:10.1016/S0025-326X(98)00162-3.

[14] US EPA *Baseline Human Health Risk Assessment Vasquez Boulevard and I-70 Superfund Site Demver, Co*; US Environmental Protection Agency: United States of America, 2001;

[15] Li, Z.; Ma, Z.; van der Kuijp, T.J.; Yuan, Z.; Huang, L. A Review of Soil Heavy Metal Pollution from Mines in China: Pollution and Health Risk Assessment. *Sci. Total Environ.* **2014**, *468–469*, 843–853, doi:10.1016/j.scitotenv.2013.08.090.

[16] BQTSB (Beijing Quality and Technology Supervision Bureau) Environmental Site Assessment Guideline; DB11/T 656-2009 2009.

[17] US EPA Human Health Evaluation Manual. In *Risk Assessment Guidance for Superfund*; Office of Emergency and Remedial Response, U.S. Environmental Protection Agency: Washington DC, United States, 1989; Vol. 1 ISBN EPA/540/1-89/002.

[18] Barnes, D.G.; Dourson, M.; Dourson, M.; Preuss, P.; Barnes, D.G.; Bellin, J.; Derosa, C.; Engler, R.; Erdreich, L.; Farber, T.; et al. Reference Dose (RfD): Description and Use in Health Risk Assessments. *Regul. Toxicol. Pharmacol.* **1988**, *8*, 471–486, doi:10.1016/0273-2300(88)90047-5.

[19] Chabukdhara, M.; Nema, A.K. Heavy Metals Assessment in Urban Soil around Industrial Clusters in Ghaziabad, India: Probabilistic Health Risk Approach. *Ecotoxicol. Environ. Saf.* **2013**, *87*, 57–64, doi:10.1016/j.ecoenv.2012.08.032.

[20] Qing, X.; Yutong, Z.; Shenggao, L. Assessment of Heavy Metal Pollution and Human Health Risk in Urban Soils of Steel Industrial City (Anshan), Liaoning, Northeast China. *Ecotoxicol. Environ. Saf.* **2015**, *120*, 377–385, doi:10.1016/j.ecoenv.2015.06.019.
